# NCAM2 Fibronectin type-III domains form a rigid structure that binds and activates the Fibroblast Growth Factor Receptor

**DOI:** 10.1038/s41598-018-27089-7

**Published:** 2018-06-12

**Authors:** Kim Krighaar Rasmussen, Maria Hansen Falkesgaard, Malene Winther, Nikolaj Kulahin Roed, Christine Louise Quistgaard, Marie Nygaard Teisen, Sofie Marie Edslev, David Leander Petersen, Ali Aljubouri, Claus Christensen, Peter Waaben Thulstrup, Leila Lo Leggio, Kaare Teilum, Peter Schledermann Walmod

**Affiliations:** 1 Biological Chemistry, Department of Chemistry, University of Copenhagen, Copenhagen, Denmark; 2 Laboratory of Neural Plasticity, Department of Neuroscience and Pharmacology, University of Copenhagen, Copenhagen, Denmark; 3 Structural Biology and NMR Laboratory, Department of Biology, University of Copenhagen, Copenhagen, Denmark

## Abstract

NCAM1 and NCAM2 have ectodomains consisting of 5 Ig domains followed by 2 membrane-proximal FnIII domains. In this study we investigate and compare the structures and functions of these FnIII domains. The NCAM1 and -2 FnIII2 domains both contain a Walker A motif. In NCAM1 binding of ATP to this motif interferes with NCAM1 binding to FGFR. We obtained a structural model of the NCAM2 FnIII2 domain by NMR spectroscopy, and by titration with an ATP analogue we show that the NCAM2 Walker A motif does not bind ATP. Small angle X-ray scattering (SAXS) data revealed that the NCAM2 FnIII1-2 double domain exhibits a very low degree of flexibility. Moreover, recombinant NCAM2 FnIII domains bind FGFR *in vitro*, and the FnIII1-2 double domain induces neurite outgrowth in a concentration-dependent manner through activation of FGFR. Several synthetic NCAM1-derived peptides induce neurite outgrowth *via* FGFR. Only 2 of 5 peptides derived from similar regions in NCAM2 induce neurite outgrowth, but the most potent of these peptides stimulates neurite outgrowth through FGFR-dependent activation of the Ras-MAPK pathway. These results reveal that the NCAM2 FnIII domains form a rigid structure that binds and activates FGFR in a manner related to, but different from NCAM1.

## Introduction

Cell adhesion molecules (CAMs) constitute a large class of plasma membrane-anchored proteins that mediate attachment of cells to neighboring cells and to the surrounding extracellular matrix. Furthermore, CAMs often form heterophilic *cis*-interactions through which they can regulate intracellular signal transduction cascades and thereby modulate a number of processes including cellular proliferation, survival, differentiation, and migration^1,2^. In addition, CAMs expressed in the nervous system can regulate processes such as neurite outgrowth, synaptic maturation and synaptic plasticity in relation to learning and memory formation^3–5^.

The Neural Cell Adhesion Molecule 2 (NCAM2) is a CAM belonging to the NCAM family, which in mammals also includes NCAM1. However, whereas NCAM1 was originally described already in the mid-1970s^6,7^ and has been extensively studied, NCAM2 was not cloned until the mid-1990’s^8^, and has only been the focus of few studies. In the nervous system NCAM2 is reported to be involved in the outgrowth and branching of neurites^9^, filopodia formation^9^, axon guidance^10^, synapse assembly/disassembly^11^, and spinal cord stem cell proliferation^12^.

The overall structure of NCAM2 is similar to that of NCAM1, with an ectodomain consisting of 5 N-terminal, membrane-distal Ig domains (denoted Ig1 to -5) followed by 2 membrane-proximal fibronectin type III domains (denoted FnIII1 and -2, with FnIII2 being closet to the cell membrane). The primary structures of the respective extracellular domains, transmembrane regions and cytoplasmic domains of NCAM1 and NCAM2 are ~36–55% identical, suggesting that the 2 proteins have related but not identical functions^10,13–15^.

The entire ectodomain of NCAM2 has been modelled using X-ray crystal structures of overlapping regions^16,17^. The structural model shows that NCAM2 molecules homodimerize *via* reciprocal interactions between the Ig1-2 domains^17^. Moreover, a domain swapping helps low affinity interaction to be more specific^16^.

The least identical region of NCAM1 and NCAM2 is the FnIII2 domain. In the NCAM1 FnIII2 domain ecto-ATP can bind to a Walker A motif, a phosphate-binding region present in many ATP and GTP utilizing proteins^18^, and binding of ATP at this site interferes with binding of the NCAM1 FnIII domains to the Fibroblast Growth Factor Receptor (FGFR). ATP thereby modulates NCAM1-FGFR-mediated intracellular signal transduction and the resulting biological responses like NCAM1-mediated neurite outgrowth^19^. Binding of ATP to NCAM1 also affects NCAM1-mediated cell adhesion^20^. A number of synthetic peptides derived from sequences in the NCAM1 FnIII domains are reported to bind and activate FGFR. Most notably, a synthetic peptide, FGL, corresponding to the NCAM1 FnIII2 FG loop has been extensively studied. This peptide promotes *e*.*g*. neurite outgrowth and neuronal cell survival *in vitro*. Furthermore, it has multiple effects *in vivo* including reduction of inflammation, promotion of learning and memory formation, and the reduction of signs of Aβ-induced neuropathology and cognitive impairment in an Alzheimer’s disease model^21^.

NCAM2 contains a Walker A motif in the FnIII2 AB loop. However, the potential ATP-binding to this site and the potential interaction between the NCAM2 FnIII domains and FGFR has not been investigated.

This study presents a nuclear magnetic resonance (NMR) spectroscopy model of NCAM2 FnIII2. Supplementary titration experiments show that ATP does not bind the Walker A motif in the NCAM2 FnIII2 domain. We show that the NCAM2 FnIII domains can induce neurite outgrowth through binding and activation of FGFR. Only 2 of 6 synthetic NCAM2-derived peptides stimulated neurite outgrowth, but at least 1 of these peptides seemed to work through binding and activation of FGFR.

## Results

### Expression and purification of recombinant proteins

To study the potential interaction between NCAM2 FnIII2 and ATP, a recombinant protein corresponding to the FnIII2 domain of human NCAM2 was expressed in the yeast *Pichia pastoris*. The domain was produced with a C-terminal His-tag, and had a total length of 108 amino acids. It was purified by affinity chromatography and gel filtration, and the purity was verified by SDS-PAGE (see Supplementary Fig. S1) and mass spectrometry (see Supplementary Fig. S2) before NMR spectra were obtained. Mass spectrometry showed that the final purified FnIII2 domain was heterogeneous with 2 or 4 extra residues originating from the signal peptide, due to inefficient Ste13 cleavage in *P*. *pastoris*. In addition to the structural analysis, the recombinant protein expressed by *P*. *pastoris* was used for *in vitro* binding studies using SPR analysis. Moreover, recombinant NCAM2 FnIII1, FnIII2, and FnIII1-2 domains were expressed in *E*. *coli*. The folding of the protein expressed in *E*. *coli* was verified by circular dichroism (CD) experiments (see Supplementary Fig. S3), and the proteins were used for SPR analysis, small-angle X-ray scattering (SAXS) experiments, as well as for cell culture studies. The main reason for changing the expression system from yeast to bacteria was to lower cost and increase yield to be able to perform more advanced scattering experiments such as contrast variation using small angle neutron scattering in the future.

### NMR structure determination and interaction studies

The NMR structure of the NCAM2 FnIII2 domain was calculated from a total of 2073 structural restraints (see Supplementary Table S1) and has been deposited to the Protein Data Bank (PDB code 2kbg). The experimental details of the structure are given in Supplementary Information (Table S1, Figures S4, S5 and S6). Not surprisingly, the structure is highly similar to the X-ray structure (r.s.m.d. = 0.95 Å using backbone atoms I^8^-F^91^ of the NCAM2 FnIII2 NMR ensemble against the same domain determined in the context of a longer ectodomain construct (PDB code: 2jll)). When comparing the NMR structure with the X-ray structure it is evident that a loop (K^42^-D^46^) is missing in the X-ray structure. This loop is well characterized in the NMR structure, and has a significant influence on the SAXS analysis. However, the overall topologies of the NMR and X-ray structures are identical, with a β-sandwich formed by two anti-parallel β-sheets consisting of 3 and 4 β-strands, respectively. One β-sheet is composed of β-strands A (I^8^-S^14^), B (S^17^-I^22^), and E (H^59^-L^62^). The other β-sheet is composed of β-strands C’ (L^49^-Q^54^), C (L^33^-S^41^), F (Y^71^-A^78^), and G (T^87^-F^91^) (see Supplementary Fig. S4b).

**Figure 1:**
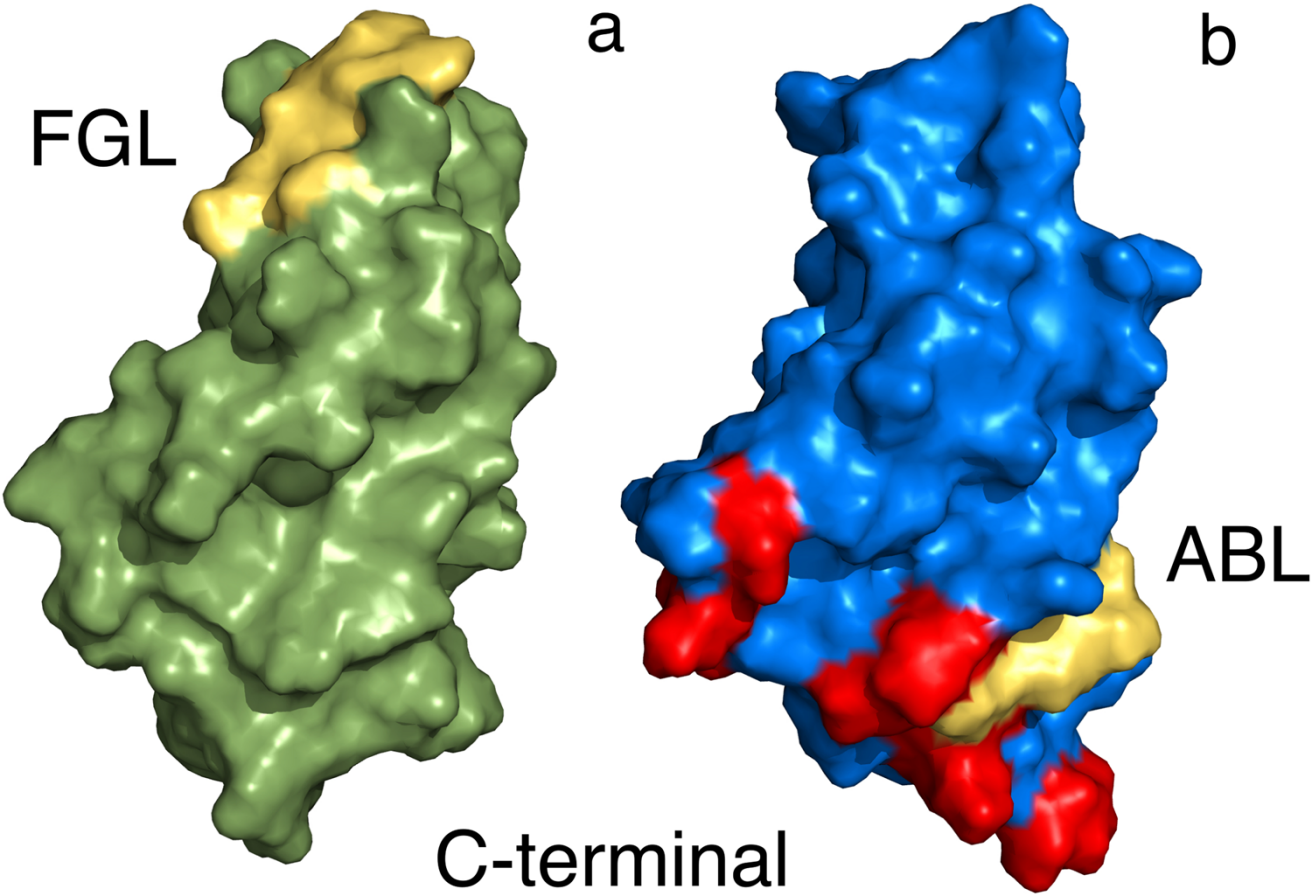
The Walker A motif is located differently in NCAM1 and NCAM2. The figure shows the locations of the Walker A motif in the FnIII2 domains of NCAM1 ((**a**) PDB code: 1lwr) and NCAM2 ((**b**) PDB code: 2kbg). The domains are presented as surface models, and are oriented in a manner that enables the visualization of the Walker A motifs, which are located differently in the two proteins. Moreover, the domains are oriented with the membrane-proximal end facing down. FGL and ABL indicate the Walker A motif-containing loop regions of NCAM1 and NCAM2, respectively. The Walker A motifs and residues perturbed when titrating NCAM2 FnIII2 with AMP-PCP are colored yellow and red, respectively.

The FnIII2 domain of NCAM2 is similar (r.m.s.d = 1.37 Å using same part of the NCAM2 FnIII2 ensemble as above) to the corresponding FnIII2 domain from NCAM1 (PDB code: 1lwr). However, the locations of the Walker A motifs are different in the 2 domains (Fig. 1); in NCAM1 FnIII2 the motif is located in the membrane-distal FG loop (amino acid residues 77–84) whereas the motif in NCAM2 FnIII2 is located in the membrane-proximal AB loop (amino acid residues 10–17).

To determine if NCAM2 FnIII2 can bind AMP-PCP, a non-hydrolysable ATP-analogue, *via* the Walker A motif, we measured ^15^N-HSQC on free NCAM2 FnIII2 and on AMP-PCP saturated NCAM2 FnIII2. The small chemical shift differences (see Supplementary Fig. S6) revealed that AMP-PCP did not bind the Walker A motif, but interacted with residues K^42^-K^44^, D^46^-Q^47^, H^64^, Q^66^-W^67^, K^96^, N^98^ and I^99^. This suggests that the negatively-charged phosphate groups of AMP-PCP interacted with the charged residues K^42^-K^44^, D^46^, H^64^, K^96^, and that the adenosine moiety of AMP-PCP formed hydrophobic interactions with W^67^ and I^99^. When highlighted on the structure the binding surface appears at the membrane-proximal part of the FnIII2 domain, and not on the side of the domain where the Walker A motif is located (see Fig. 1). The results therefore suggest that the interaction is not biologically relevant.

### Structural analysis of NCAM2 FnIII1-2 in solution

**Figure 2:**
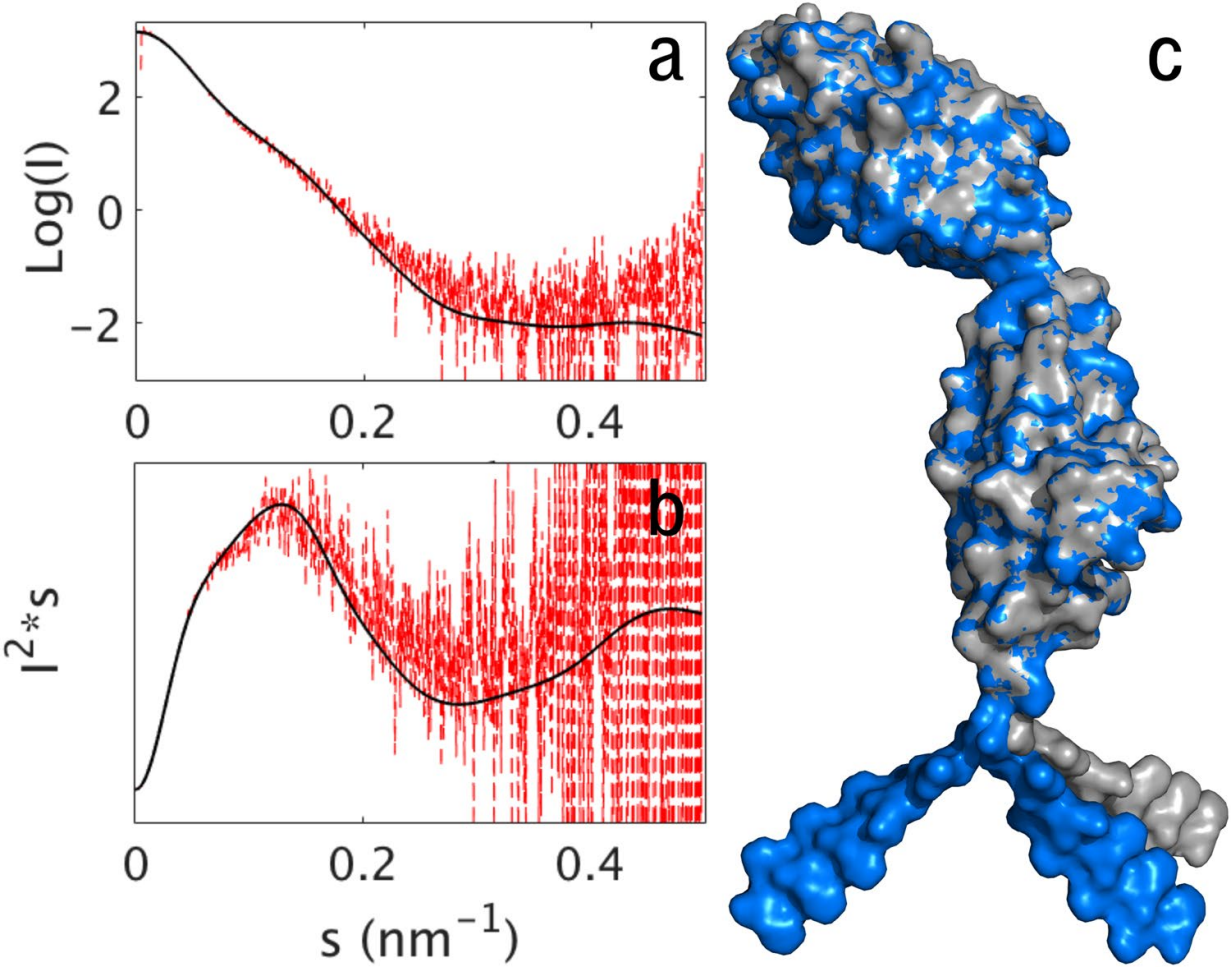
Flexibility of the NCAM2 FnIII1-2 domains. SAXS scattering data (**a**) was plotted in a Kratky plot (**b**), and revealed a multi-domain protein with flexibility. Due to flexibility, normal mode analysis was performed with SREFLEX. This resulted in 3 structural models (**c**) restrained models [blue] and unrestrained model [grey]) representing possible conformational changes improving the fit to experimental SAXS profile (**a**). According to the SREFLEX analysis the flexibility is located C-terminally with the His-tag moving freely around.

The X-ray structure of NCAM2 IgIV-FnIII2 (PDB code: 2jll) indicates a relatively static structure in the region of the FnIII domains due to the very short (two amino acid residues) linker between the FnIII1 and -2 domains. Notably, the linker contains a proline residue, which, due to its ring structure, is expected to rigidify the short linker. Moreover, the possibilities for hydrogen bonds between the FnIII1 domain and the linker could further rigidify this part of NCAM2. To investigate the structure in more detail we performed SAXS experiments (Fig. 2a), which give information of the overall shape and flexibility of proteins in solution. NCAM2 FnIII1-2 M_w_ was determined with high precision to be within 6% of the theoretical M_w_ (Table 1), and no aggregation was observed from the Guinier region (see Supplementary Fig. S7a). A CRYSOL fitting using the NCAM2 FnIII1-2 domain models from a crystal structure (PDB code: 2jll) including a model of the His-tag indeed corresponded to the measured NCAM2 FnIII1-2 SAXS data with a χ^2^-value of 0.86 (see Supplementary Fig. S7b). This indicates that the FnIII1-2 structure in solution indeed is relatively rigid. Since the region between NCAM2 FnIII1 and -2 previously, in a simulation study, has been described as flexible^17^, and the Kratky plot derived from the SAXS data presented here showed multi-domain scattering particle with flexibility (Fig. 2b) we performed SREFLEX^22^ analysis on NCAM2 FnIII1-2. This algorithm estimates flexibility by allowing for conformational changes in order to improve agreement with experimental scattering curve. The normal mode analysis resulted in a better agreement with experimental data (Fig. 2a, χ^2^ = 0.81), but the flexibility was due the C-terminal region with the His-tag (Fig. 2c). This indicates that the linker between the FnIII domains in NCAM2 should be seen as a rigid linker, and could maintain the 2 FnIII domains at a prefixed position to enhance interactions with ligands, such as an NCAM2-FGFR interaction.

**Table 1:** Data from SAXS analysis of NCAM2 FnIII1-2 and bovine serum albumin (BSA).

	*R*_g_^a^ (nm)	*R*_g_^b^ (nm)	*V*_p_^c^ (nm^3^)	*M*_wExp_^d^ (kDa)	*M*_wExp_^e^ (kDa)	*M*_w_^f^ (kDa)
*NCAM*2*FnII1*-*2*	3.18	3.31	32.96	23.48	23.64	22.43
*BSA*	3.08	3.14	117.80	67.32	67.67	66.46

^a^Radius of gyration, *R*_g_, estimated from the Guinier approximation.

^b^*R*_g_ estimated by AutoGNOM.

^c^The excluded hydrated volume (Porod volume), *V*_p_.

^d^Molecular weight, *M*_w_, estimated from *I*(0) obtained by the Guinier approximation.

^e^*M*_w_ estimated from AutoGNOM.

^f^Theoretical *M*_w_.

### NCAM2 FnIII domains interact with FGFR1

Since NCAM1 FnIII domains have been shown to interact *in vitro* with FGFRs^19,23^, we tested whether human NCAM2 FnIII domains and FGFR1 could also interact *in vitro*.

**Figure 3:**
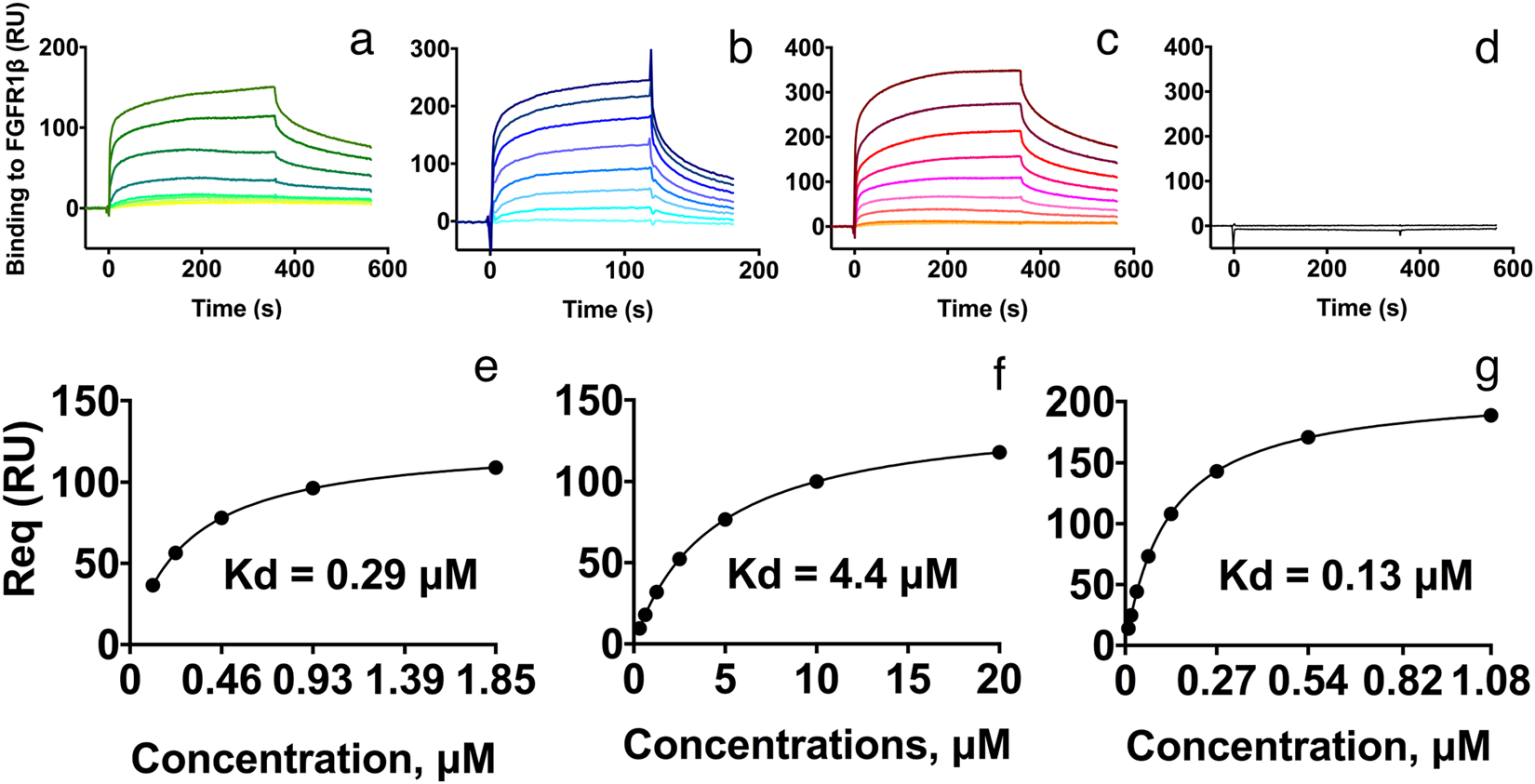
NCAM2 FnIII domains bind to FGFR1. Surface plasmon resonance analysis was utilized to investigate interactions between the NCAM2 FnIII1, FnIII2, and FnIII1-2 domains and the ectodomain of FGFR1. Approximately 1000 resonance units (RUs) of the FGFR1β ectodomain (Ig2-Ig3) were immobilized on a sensor chip and recombinant NCAM2 FnIII1 (**a**), FnIII2 (**b**) and FnIII1-2 (**c**) were injected at the indicated concentrations. Binding is expressed as the response difference between the binding of the respective recombinant proteins to a cell on the sensor chip with the immobilized FGFR1β and a control cell on the same sensor chip coated with IgG (**d**). On the basis of the presented steady state affinity fit curves, *K*_*d*_ values of 0.29 (χ^2^ = 0.057), 4.4 (χ^2^ = 0.016) and 0.13 (χ^2^ = 0.028) μM were estimated for NCAM2 FnIII1 (**d**), FnIII2 (**e**) and FnIII1-2 (**f**), respectively.

Surface plasmon resonance (SPR) analysis demonstrated a concentration-dependent binding between FGFR1β immobilized on the chip, and NCAM2 FnIII domains in solution (Fig. 3). On the same chip an Ig domain had been immobilized as a negative control to detect unspecific binding. No unspecific binding was observed for any of the tested FnIII domains (Fig. 3). By steady state fitting we roughly estimated the *K*_*d*_’s for the interactions between FGFR1β and the NCAM2 FnIII domains. The *K*_*d*_’s were all in the µM range (Fig. 3), which corresponds to *K*_*d*_ values obtained for other FGFR-FnIII domains interactions^23^. *K*_*d*_’s obtained from NCAM2 FnIII1-2 domains expressed from *P*. *pastoris* or in *E*.*coli* were in similar range 0.4 µM with χ^2^ = 0.061 (Supplementary Fig. S8) and 0.13 µM with χ^2^ = 0.028 (Fig. 3), respectively. Thus, the *K*_*d*_ for what we see as the biologically binding partner to FGFR, was reproducible.

### NCAM2 FnIII domains stimulate neurite outgrowth

**Figure 4:**
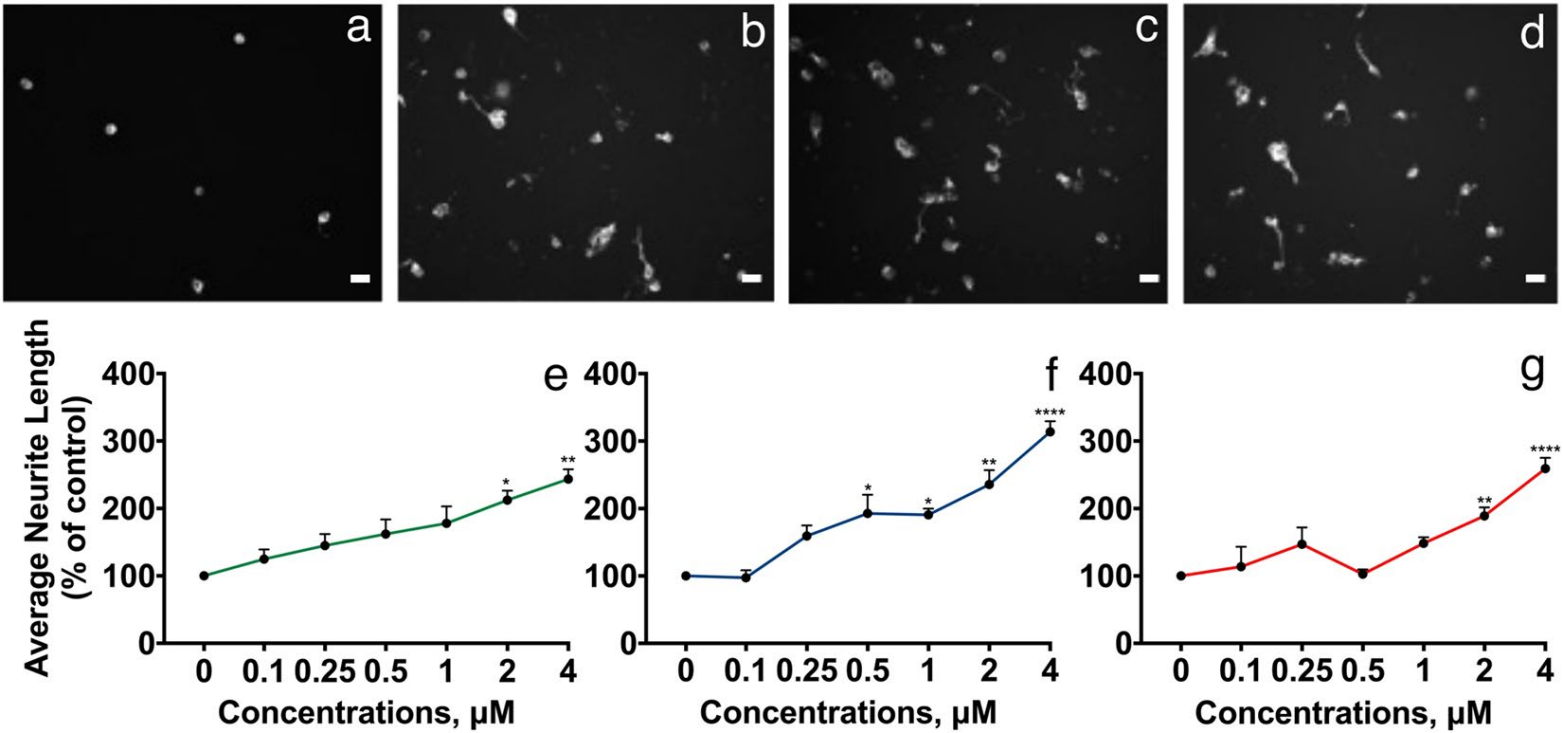
NCAM2 FnIII1, FnIII2 and FnIII1-2 domains induce neurite outgrowth. CGNs were grown for 24 h on a coat of different concentrations of recombinant NCAM2FnIII-domains. The neurons were immunostained and neurite lengths were estimated by a stereological approach from the recorded micrographs. Top panel: Representative micrographs showing CGNs grown on 0 μM protein (control, **a**), and 4 μM NCAM2 FnIII1 (**b**), FnIII2 (**c**), and FnIII1-2 (**d**), respectively. (**e**–**g**) Concentration-response curves for normalized average neurite length vs. concentration of NCAM2 FnIII1 (**e**), NCAM2 FnIII2 (**f**), and NCAM2 FnIII1-2 (**g**), respectively. Before cells were plated the wells were coated with 7 concentrations of the respective NCAM2 FnIII domains (0, 0.3125, 0.625, 1.25, 2.5, 5.0, 10.0 µg/cm^2^). Data have been normalized to the negative control (0 μM protein) and are shown as mean and SEM (*n* = 3–4). The individual concentration-response data were analyzed separately by one-way ANOVA for repeated measures followed by Tukey’s multiple comparison test. All 3 proteins significantly induced neurite outgrowth. NCAM2 FnIII1: F (6, 21) = 3.472, *p* < 0.0153. NCAM2 FnIII2: F (6, 12) = 18.72, *p* < 0.0001. NCAM2 FnIII1-2: F (6, 12) = 13.94, *p* < 0.0001. *^,^** and ***p < 0.05, 0.01, and 0.001, respectively, when compared to the negative control. Scale bars = 10 µM. The total average neurite length per cell for domains can be found in Supplementary Table S2.

To investigate the biological function of the NCAM2 FnIII domains, we investigated neurite outgrowth in response to exposure to the FnIII domains. Rat cerebellar granule neurons (CGNs) were seeded on Permanox in the absence or presence of NCAM2 FnIII domains coated in different concentrations. As seen from micrographs in Fig. 4a–d FnIII1, FnIII2, and FnIII1-2 all induced neurite outgrowth at a coating concentration of 4 µM, when compared to the negative control. Our negative control does not induce neurite outgrowth. Previous studies performed with BSA, also shows that it does not induce neurite outgrowth^24^. Figure 4e–g presents concentration-response curves of the average neurite lengths in response to different concentrations of the respective FnIII constructs. FnIII1, FnIII2, and FnIII1-2 all statistically significantly induced neurite outgrowth in a concentration-dependent manner. Surprisingly, no synergistic effect was observed for the combination of FnIII1 and -2. Thus, the single domains and the double domain construct induced neurite outgrowth with apparently similar potencies and efficacies (Fig. 4e–g).

To investigate the signaling pathway involved in the neurite outgrowth induced by the FnIII domains experiments were performed, where neurite outgrowth induced by FnIII1-2 was estimated in response to treatment with different concentrations of an FGFR inhibitor (SU5402) and a MEK 1/2 inhibitor (U0126).

**Figure 5:**
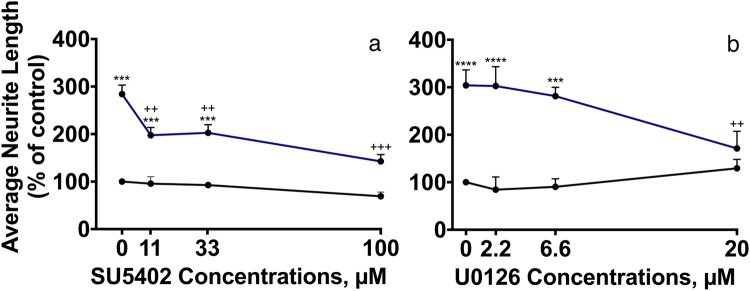
NCAM2 FnIII1-2 activates signaling through FGFR and MEK. CGNs were treated for 24 h with different concentrations of the FGFR kinase inhibitor SU5402 (Sigma Aldrich; **a**) or the MEK inhibitor U0126 (MERCK; **b**) in the absence (black curves) or presence (blue curves) of 4 μM NCAM2 FnIII1-2. The inhibitors were added to the wells 10 min after the cells had been plated. In each experiment, 4 concentrations were used per inhibitor: 0, 11.1, 33.3 and 100 μM (for SU5402), and 0, 2.2, 6.6 and 20 μM (for U0126). Average neurite lengths were estimated by a stereological approach from recorded micrographs. Data are shown normalized to control (0 μM inhibitor in the absence of FnIII1-2) as mean and SEM (*n* = 3). The individual concentration-response data were analyzed by one-way ANOVA for repeated measures followed by Tukey’s multiple comparison test. (**a**) F (12, 35) = 36.55, *p* < 0.0001. (**b**) F (12, 35) = 28.14, *p* < 0.0001. *^,^** and ***p < 0.05, 0.01, and 0.001, respectively, when compared to negative control (no FnIII1-2, 0 μM inhibitor). ^+,++^ and ^+++^*p* < 0.05, 0.01, and 0.001, respectively, when compared to positive control (FnIII1-2, 0 μM inhibitor). The effect of the total average neurite length per cell for domains cultured with inhibitor can be found in Supplementary Table S2.

Figure 5a shows that SU5402 statistically significantly inhibited the neurite outgrowth induced by FnIII1-2 by ~50% already at a concentration of 11 μM. However, higher concentrations of SU5402 had only minor additional inhibitory effects. These data suggest that neurite outgrowth induced by NCAM2 FnIII1-2 in part, but not exclusively is mediated through activation of FGFR.

Figure 5b shows that U0126 statistically significantly inhibited the neurite outgrowth induced by FnIII1-2 in a concentration-dependent manner to a level not significantly different from the negative control (cell cultured in the absence of FnIII1-2 and U0126). These data indicate that the neurite outgrowth induced by NCAM2 FnIII1-2 is mediated by signaling through the Ras-MAPK pathway. The total average neurite lengths in µm and standard error of means (SEMs) can be found in Supplementary Table S2.

### Sequence-based investigations for an NCAM2 FnIII1-2-FGFR interaction site

**Figure 6:**
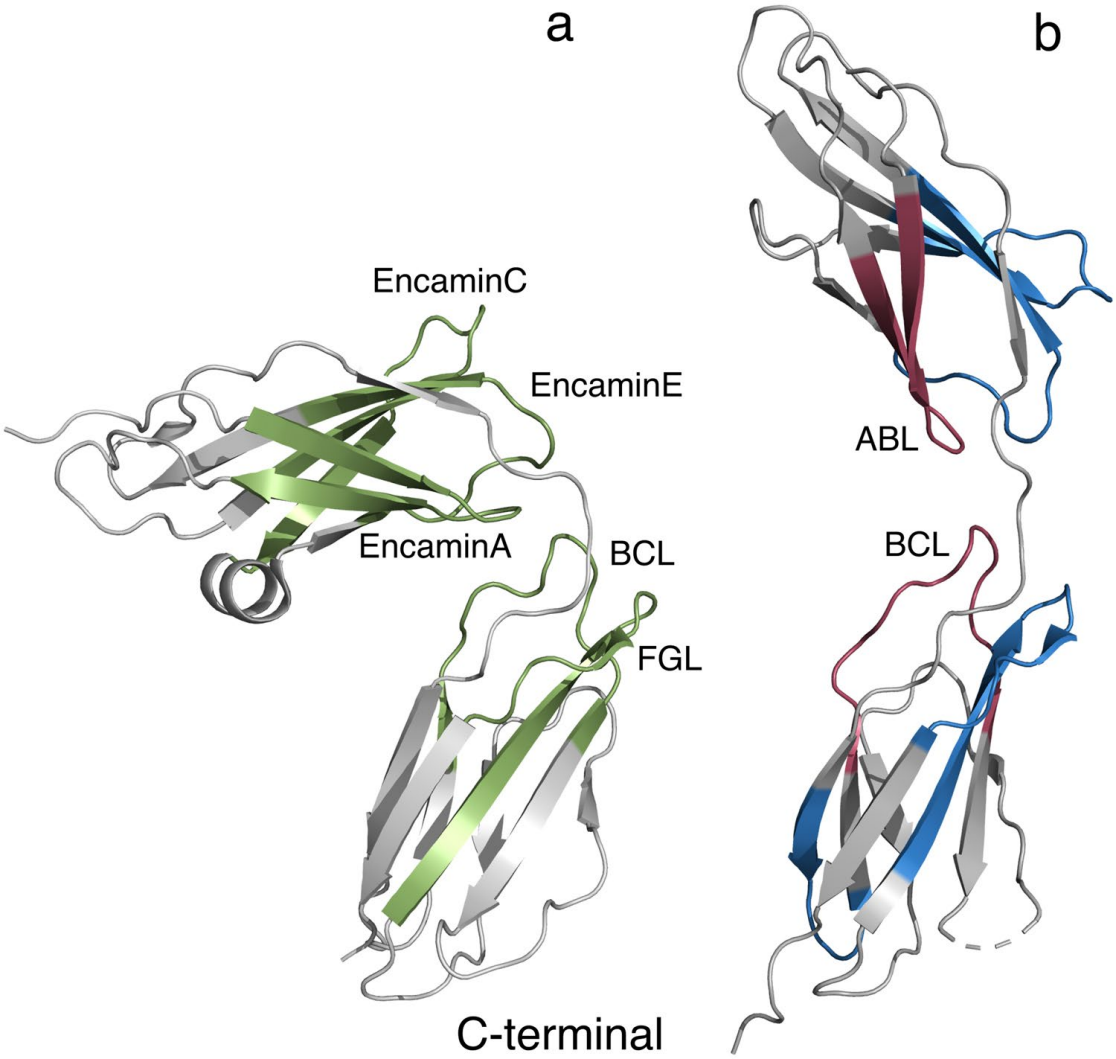
Structural comparison of the NCAM1 and NCAM2 FnIII domains. The FnIII domains (grey) from NCAM1 ((**a)** PDB code: 2vkw) and NCAM2 ((**b)** PDB code: 2jll) were aligned to compare secondary structure differences. The domains are oriented with the membrane-proximal end facing down. Pairwise, the domains have same overall topology. However, NCAM1 FnIII1 contains an α helix between the D and E strands not observed in NCAM2 FnIII1. Biologically active peptides derived from NCAM1 FnIII1-2 (EncaminA, -C, -E, BCL and FGL) are colored in green. All NCAM1 FnIII1-2 peptides are in the literature reported to interact with FGFR^53^. Peptides derived from NCAM2 FnIII1-2, and used in this study are colored red (biologically active, ABL and BCL) and blue (not biologically active). When comparing the regions of NCAM1, from which the respective peptides are derived, with the corresponding regions in NCAM2, they all have highly identical secondary structures.

Since the FnIII1-2 domains bind FGFR (Fig. 3) and in part stimulate neurite outgrowth by activation of FGFR (Fig. 5a) we tried to identify regions of high sequence conservation between NCAM1 FnIII1-2, and NCAM2 FnIII1-2. In particular, we focused on the sequences of synthetic peptides derived from NCAM1 reported to stimulate neurite outgrowth through binding and activation of FGFR. In the literature 5 NCAM1 peptides (EncaminA (FnIII1), EncaminC (FnIII1), EncaminE (FnIII1), BCL (FnIII2), and FGL (FnIII2) are reported to bind and activate FGFR^19,25,26^. Each peptide corresponds to a structural motif consisting of 2 adjacent β-strands and the loop between them, as highlighted in green on the structure of NCAM1 FnIII1-2^27^ shown in Fig. 6a, and the sequence alignment in Fig. S9. From the sequence alignments, it is evident that 4 out of the 5 NCAM1-derived peptides (EncaminA, EncaminC, EncaminE and BCL) are conserved between human NCAM1 and NCAM2 in the loop regions with respect to amino acids properties such as hydrophobicity, polarity, and charge (see Supplementary Fig. S9).

The sequences corresponding to the Encamin peptides are 23–35% conserved between the 2 proteins. The BCL peptide (NLIKQDDGGSPIRHY) derived from the NCAM1 FnIII2 domain is the peptide where the sequence is most highly conserved between NCAM1 and NCAM2 FnIII2 domains (60%), and this sequence is also highly conserved across the selected species. The glutamine residue in position 5 of BCL is essential for the function of the peptide. Thus, a Q → A substitution completely abrogates the function of the peptide^26^. Interestingly, this glutamine residue is conserved between NCAM1 and -2. The least conserved loop region between NCAM1 and NCAM2 FnIII2 domains (40%) is found in the sequences corresponding to the FGL peptide (EVYVVAENQQGKSKA). Substitution of the 2 glutamine residues (polar uncharged) at positions 9–10 to alanine residues totally abrogates the neuritogenic effect of FGL^19^. In the corresponding region of NCAM2 FnIII2 the 2 glutamine residues are replaced with an arginine residue (charged) and a leucine residue (hydrophobic) (see Supplementary Fig. S9).

### Synthetic peptides derived from NCAM2 FnIII1 and FnIII2 activate FGFR

Based on the sequence analysis described above we designed peptides derived from sequences in the NCAM2 FnIII1-2 domains, but corresponding to NCAM1 EncaminA, -C and -E (peptides derived from the FnIII1 domain, denoted FnIII1-AB, FnIII1-CD and FnIII1-EF), BCL and FGL (derived from the FnIII2 domain, denoted FnIII2-BC and FnIII2-FG respectively). We also designed a peptide from the FnIII2 AB loop (FnIII2-AB), since this region contains the potential Walker A motif. Like the NCAM1-derived peptides, the NCAM2-derived peptides were synthesized as tetramers linked to a lysine backbone, thus enabling clustering of potential binding partners.

**Figure 7:**
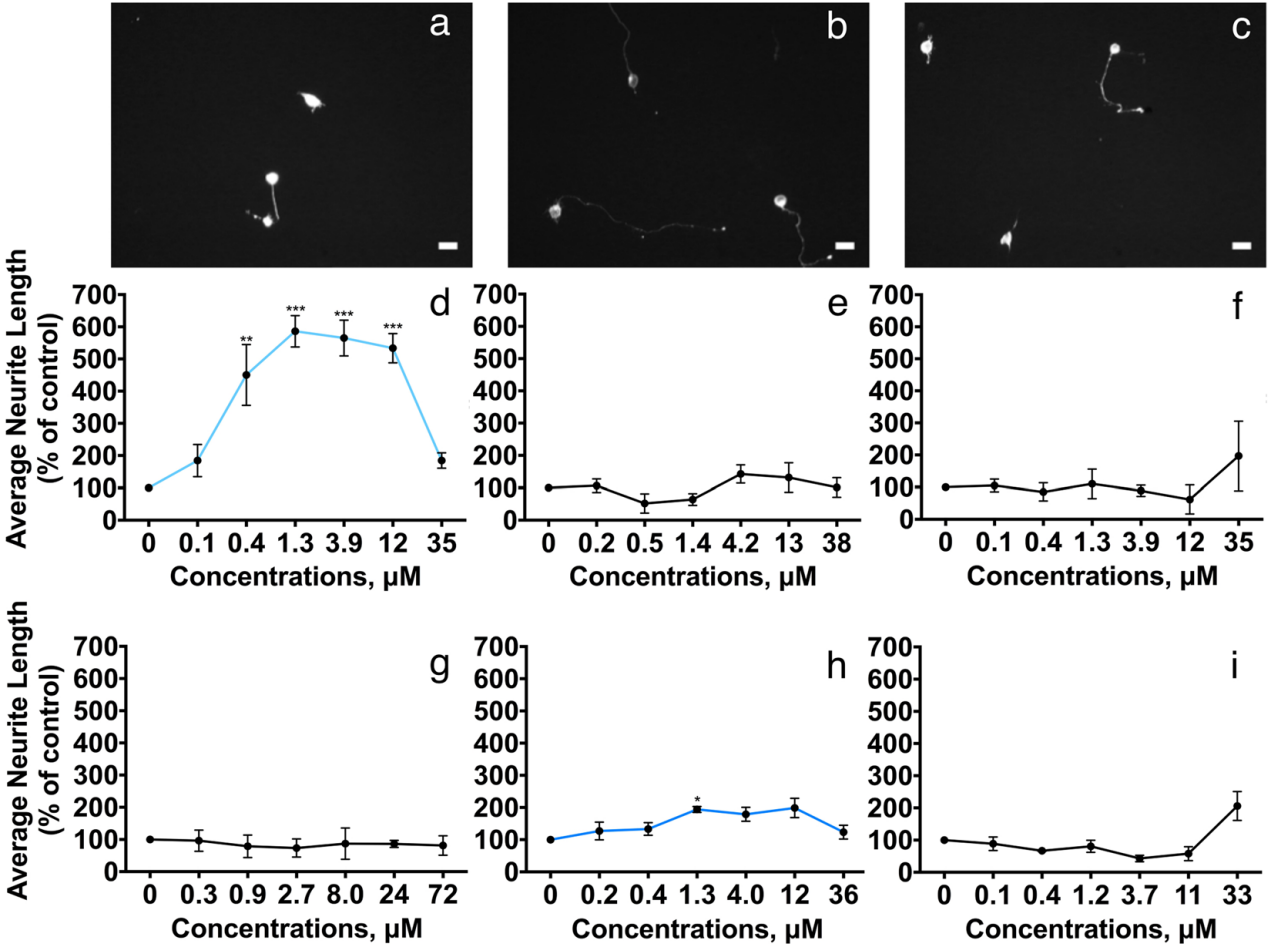
Neurite outgrowth induced by synthetic peptides derived from NCAM2 FnIII1 and FnIII2. CGNs were treated with dendrimeric peptides at the indicated concentrations for 24 h. The neurons were immunostained and average neurite lengths were estimated by a stereological approach from recorded micrographs. Top panel shows representative micrographs of CGNs: not stimulated (control) (**a**), stimulated with NCAM2-FnIII1-AB (**b**), and NCAM2-FnIII2-BC (**c**). Graphs d-f show data for peptides derived from sequences in NCAM2 FnIII1, and graphs g-i show data for peptides derived from sequences in NCAM2 FnIII2. The graphs present concentration-response curves for average neurite length vs. the concentration of the respective peptides. Data were normalized to the negative control (0 μM peptide) and are shown as mean and SEM (*n* = 3). The individual concentration-response data were analyzed by one-way ANOVA for repeated measures followed by Tukey’s multiple comparison test. FnIII1-AB: F (6, 12) = 20.45, *P* < 0.0001. FnIII1-CD: F (6, 12) = 1.056, P = 0.4384. FnIII1-EF: F (6, 6) = 0.6812, P = 0.6736. FnIII2-AB: F (6, 12) = 0.5348, P = 0.7723. FnIII2-BC: F (6, 12) = 3.944, P = 0.0207. FnIII2-FG: F (6, 12) = 2.844, P = 0.0583. *^,^** and ***p < 0.05, 0.01, and 0.001, respectively, when compared to control. Scale bars = 10 µm. The total average neurite length per cell for peptides can be found in Supplementary Table S3.

The biological activities of the designed peptides were tested by performing concentration-response evaluations of their abilities to stimulate neurite outgrowth in primary cultures of CGNs (Fig. 7). Only 1 peptide from the FnIII1 domain, FnIII1-AB, corresponding to EncaminA in NCAM1, had significant effects on neurite outgrowth. This peptide demonstrated a statistically significant bell-shaped, concentration-dependent stimulation of neurite outgrowth with a maximal stimulation of ~600% at a concentration of 1.3 μM (Fig. 7d). Additional peptides derived from the FnIII1 domain, FnIII1-CD and FnIII1-EF, corresponding to EncaminC and -E from NCAM1, had no significant effects on neurite outgrowth within the tested concentration ranges (Fig. 7e,f).

Of the 3 peptides derived from NCAM2 FnIII2 only 1 peptide, FnIII2-BC (corresponding to the BCL peptide from NCAM1), had moderate, but significant effects on neurite outgrowth. This peptide induced a statistically significant, bell-shaped, concentration-dependent stimulation of neurite outgrowth with a maximal stimulation of ~200% at a concentration of 1.3 μM (Fig. 7h). The 2 peptides FnIII2-AB (containing the Walker A motif) and FnIII2-FG (corresponding to the FGL peptide derived from NCAM1) had no statistically significant effects on neurite outgrowth within the tested concentration ranges (Fig. 7g,i). FnIII2-BC unfortunately demonstrated a high degree of variability in the biological effects, and consequently it was necessary to exclude this peptide from further investigations. However, since FnIII1-AB represented a potential FGFR binding site in NCAM2, it was decided to investigate whether the neuritogenic effect of this peptide was mediated through FGFR.

### Dendrimeric FnIII1-AB induces neurite outgrowth through FGFR and MEK1/2

Experiments were conducted, where CGNs grown in the absence or presence of FnIII1-AB were stimulated with different concentrations of the FGFR kinase inhibitor SU5402 or the MEK1/2 kinase inhibitor U0126.

**Figure 8:**
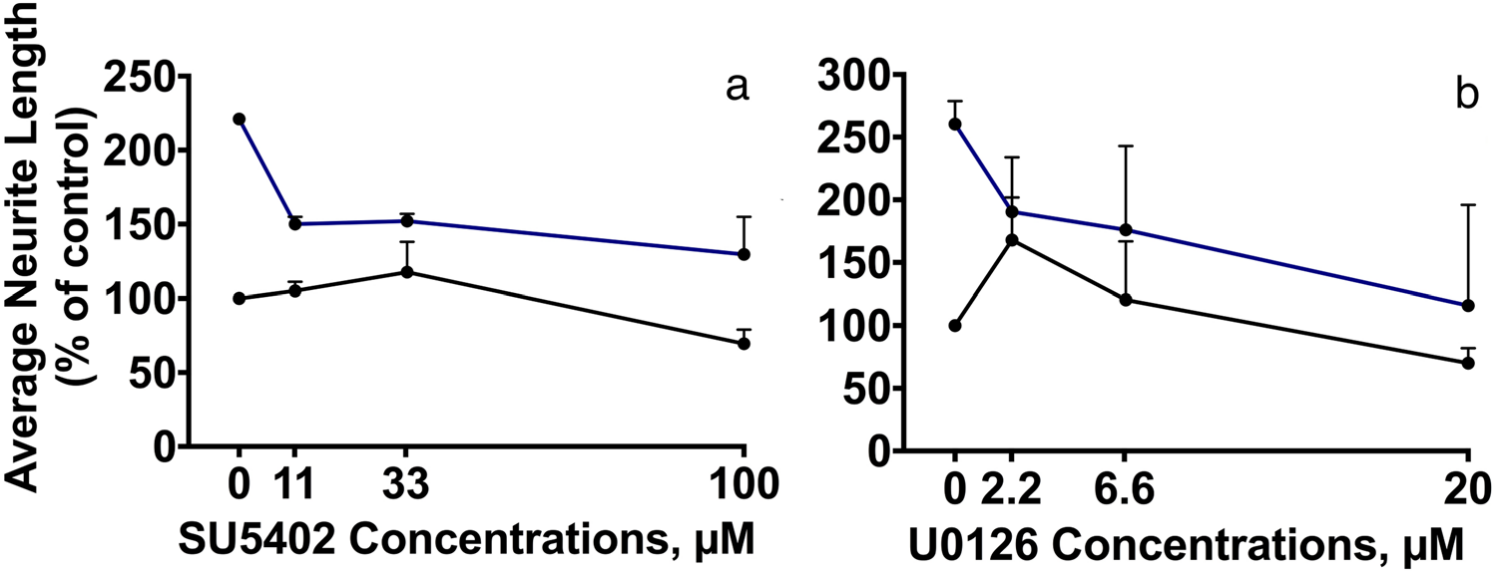
Dendrimeric FnIII1-AB induce neurite outgrowth through FGFR and MEK1/2. CGNs were treated for 24 h with different concentrations of the FGFR kinase inhibitor SU5402 (**a**) or the MEK inhibitor U0126 (**b**) in the absence (black curves) or presence (blue curves) of 3.9 μM dendrimeric FnIII1ab. Average neurite lengths were estimated by a stereological approach from recorded micrographs. Data were normalized to the negative control (0 μM inhibitor in the absence of peptide) and are shown as mean and SEM (*n* = 2). The effect of the total average neurite length per cell for FnIII1-AB cultured with inhibitor can be found in Supplementary Table S3.

The dose-response data for the experiments with the 2 inhibitors are shown in Fig. 8. Already at a concentration of 11 μM SU5402 inhibited FnIII1-AB-induced neurite outgrowth by more than 50% (Fig. 8a). However, higher concentrations of SU5402 did not inhibit the response further. Dose-response experiments with U0126 demonstrated that the neurite outgrowth-stimulating effect of FnIII1-AB could be almost entirely inhibited even at low concentrations of the inhibitor (Fig. 8b).

These results suggest that FnIII1-AB to a large extent, but not exclusively, stimulated neurite outgrowth through activation of FGFR, and that the response requires activation of the Ras-MAPK pathway. The total average neurite lengths in µm and SEMs can be found in Supplementary Table S3.

## Discussion

Walker A motifs are associated with phosphate binding, and have the general pattern A/G-XXXX-GK-S/T with X being an arbitrary amino acid residue. Especially the lysine residue and the main chain are crucial for nucleotide binding^28^. The motif is present in many ATP and GTP utilizing proteins such as ATP synthase and myosin, but is also found *e*.*g*. in NCAM1, where binding of ecto-ATP to the motif abrogates NCAM1-FGFR interactions^19,29^. Using NMR we here show that the Walker A motif in the NCAM2 FnIII2 domain does not bind the ATP analogue AMP-PCP (Fig. S6).

In NCAM1 FnIII2 the motif is located in the distal part of the domain, whereas it in NCAM2 FnIII2 is located close to the membrane (Fig. 1). The fact that both proteins do have a Walker A motif is therefore not the result of sequence homology, and therefore it is not surprising, that the functions of the Walker A motifs in the two proteins are not conserved. The interaction between NCAM1 and the ATP analogue involves two lysine residues and a tyrosine residue, and it has been hypothesized that the adenine ring of ATP makes π-π stacking with the aromatic tyrosine ring, while the negatively charged phosphate groups interact with the two positively charged lysine side-chains in at ‘fork’-like manner^19^. It is clear, when comparing the two primary structures, that NCAM2 lacks an extra lysine and an aromatic residue. Therefore, it seems likely that these residues are crucial to the interaction, and could be the reason for the lack of NCAM2 AMP-PCP interaction at the Walker A motif. Indeed the Walker A motif in NCAM2 may have no function, since a synthetic peptide derived from this region of NCAM2 also failed to demonstrate any biological activity (Fig. 7d).

The primary structure of the FnIII2 FG loop is poorly conserved between NCAM1 and NCAM2, whereas the tertiary structures are very similar as seen from the alignment of NCAM1- and NCAM2 FnIII2 (Fig. 6). In NCAM1 the FnIII2 FG region is important both for ATP binding and NCAM1-FGFR interactions^19^, whereas this is not the case for NCAM2, where a synthetic peptide derived from this region demonstrated no biological activity (Fig. 7f). Hence, it is the specific sequence or the amino acid composition of the NCAM1 FnIII2 FG loop, and not its position relative to the membrane or its tertiary structure, that is important for its ability to bind FGFR. Consistent with this interpretation, two amino acid residues (QQ) vital for the binding of this region of NCAM1 to FGFR^19^ are missing in NCAM2.

The functional differences between the FnIII domains of NCAM1 and NCAM2 are supported by SPR analyses and biological studies. Thus, the *K*_*d*_ values for the binding of the FnIII1-2 domains of NCAM1 and NCAM2 to the Ig2-3 domains of FGFR1 are in the order of 10 µM^19^ and 0.13 µM (Fig. 3), respectively. In neurite outgrowth experiments, recombinant NCAM2 FnIII2 has prominently stronger effects on average neurite outgrowth than recombinant NCAM1 FnIII2. Thus, the NCAM1 domain could only stimulate neurite outgrowth of CGNs to an average neurite length of ~165% of the negative control^19^, whereas the NCAM2 domain stimulated neurite outgrowth to an average neurite length of ~300% of the negative control (Fig. 4). These differences should not be over interpreted, but an ability of NCAM2 to bind FGFR more strongly than NCAM1 would be a way to compensate for the generally higher number of NCAM1 molecules in neurons (according to www.genecards.org^30^ the estimated level of NCAM1 protein in the nervous system is in the order of 10 times higher than that of NCAM2).

Inspecting the structural model of the NCAM2 ectodomain^17^, a different interaction mechanism between FGFR and FnIII1-2 domains of NCAM1 and NCAM2 makes sense, since a glycosylation site located in NCAM2 FnIII1 (but not in NCAM1 FnIII1) might causes steric hindrance for an interaction between the NCAM2 FnIII2 FG loop and FGFR by covering the FG loop^17^.

The observed rigidity of the NCAM2 FnIII1-2 double domain could be a way to predefine the distance for an NCAM2 *trans*-interaction and hence the cell-cell distance in NCAM2-mediated cell adhesion. It could also be a way to facilitate NCAM2-FGFR interactions, by making the NCAM2 FnIII1 domain easily accessible to FGFR.

Synthetic peptides were designed to map interaction sites between the NCAM2 FnIII domains and FGFR1. From the experiments we identified 2 peptides, which could induce neurite outgrowth, one from the FnIII1 domain and one from the FnIII2 domain. The peptide from FnIII1 was more potent than the peptide from FnIII2 (Fig. 7). This difference correlates with the measured *K*_*d*_ affinities for the binding of the respective FnIII domains to FGFR1 (0.29 µM and 4.4 µM; Fig. 3) and further supports the notion that a different interaction mechanism exists for the respective binding of NCAM1 and NCAM2 to FGFR. However, it should be emphasized that the NCAM2-derived peptides tested in this study do not cover all regions of the NCAM2 FnIII domains. Additional FGFR binding sites with potentially higher affinities may exist in the NCAM2 FnIII domains.

The peptide studies indicate that NCAM2 in part binds FGFR *via* the AB loop in FnIII1, and to a lesser extent via the BC loop in FnIII2, an observation that makes sense, since these regions spatially are in close proximity to each other (Fig. 6b).

In conclusion, the data demonstrate that ecto-ATP does not bind NCAM2 FnIII2, but that the NCAM2 FnIII domains do bind and activate FGFR. The function of the NCAM2-FGFR interaction *in vivo* should be investigated in future studies.

## Materials and Methods

### Protein expression and purification

Recombinant human NCAM2 FnIII1 and FnIII1-2 (amino acid residues 498–591 and 498–693, respectively; UniProtKB identifier: O15394) were expressed in *E*. *coli* and refolded and purified using the strategy previously described^31^. Eluted proteins were further purified by size exclusion chromatography as described below; with the exception that pH was 7.4. For NMR the recombinant NCAM2 FnIII2 (amino acid residues 592–693; UniProtKB identifier: O15394), here numbered amino acid residues 1–102, was expressed in the yeast *P*. *pastoris*^16^. The protein contained an N-terminal sequence (EAEASM), derived from the multiple cloning site of the pPICZaC vector (Invitrogen), and a C-terminal 6xHis-tag. The FnIII2 encoding vector was transformed into the KM71H strain and expressed according to the manufacturer’s instructions (Invitrogen). The recombinant protein was purified by affinity chromatography using a Ni^2+^-NTA resin (QIAGEN) followed by gel filtration using a HiLoad 16/60 XK 16 Superdex-75 prep grad column (GE Healthcare) and elution in an NMR buffer containing 20 mM Na_2_PO_4_/NaH_2_PO_4_ buffer, pH 6.5.

### NMR measurements

The following samples were used to record NMR spectra: 1.4 mM uniform ^13^C^15^N-labeled NCAM2 FnIII2 expressed from *P*. *pastoris* using ^13^C-methanol as carbon source (solubilized in H_2_O or D_2_O). For the assignment of the backbone atoms the following NMR spectra were recorded; ^15^N-HSQC in H_2_O or D_2_O, HNCACB, CBCA(CO)NH, HNCO, HN(CA)CO, HNCA and HN(CO)CA. To obtain side chain assignments the following additional spectra were recorded: CCONH, HCCONH, ^15^N-TOCSY-HSQC (50 ms), and HCCH-TOCSY (14 ms) for aliphatic and aromatic regions. The NOEs were obtained from the following NOESY spectra: ^15^N-NOESY-HSQC (150 ms) in H_2_O and ^13^C-NOESY-HSQC (100 ms) in D_2_O for aliphatic and aromatic regions. All spectra were recorded using the standard setup provided by the ProteinPack (Varian Inc.). The spectra were processed by the NMRPipe program^32^ and analyzed by the Ccpnmr program package^33^. The NMR experiments were all performed using either Varian Unity Inova 750 or 800 MHz spectrometer at 298 K.

### Structure calculations

The initial structural model and automatic NOE assignments were performed using the CYANA.2.1 programme^34^. The automatic NOE assignment tolerance was set at 0.025-, 0.025- and 0.45 ppm for indirect ^1^H-, direct ^1^H-, and ^13^C or ^15^N dimensions, respectively. The upper limit values were set to 2.4–6.0 Å. Random seed was set to 434726. Subsequently, the 3D *de novo* structure model was recalculated for refinement using a simulated annealing protocol using the XPLOR-NIH program^35,36^. A total of 1934 NOE restrains were derived from the 100 ms ^13^C-NOESY-HSQC and the 150 ms ^15^N-NOESY-HSQC using the same upper limits (increased by 0.5 Å if needed). From the TALOS Programme^37^ 59 additional torsion angle constrains were determined and applied to the structure calculation. In addition, 21 hydrogen bonds were estimated using MOLMOL^38^ with the criteria that each H bond is located in all 20 structure models, and has a maximum distance of 2.4 Å with a 35° angle. The rate of hydrogen exchange for each amide was quantified by measuring ^15^N-HSQC spectra at t = 0 min, 60 min, and 24 h. after dissolving 1.4 mM NCAM2 FnIII2 in D_2_O to verify the presence of hydrogen bonds, which were included as restraints in the structure calculations. The structural model was calculated using Xplor-NIH and analyzed and checked using the MOLMOL and PROCHECK_NMR programs^39^. Figures were prepared using MOLMOL or PyMOL (http://www.pymol.org).

### Small angle X-ray scattering (SAXS) data collection, processing and modelling

SAXS data of NCAM2 FnIII1-2 were collected at station BM29 at the European Synchrotron Radiation Facility Grenoble, France, using a PILATUS detector. Data were collected using a sample to detector distance of 2.87 m and a temperature of 277.15 K covering a range of momentum transfer of 0.05 < *s* < 5 nm^−1^, where *s* = 4πsinθ/λ, 2θ is the scattering angle and λ = 0.992 Å. Absolute intensity calibration was carried out using water. Protein concentrations were determined by measuring absorbance at 280 nm accounting for the theoretical extinction coefficient (29910 M^−1^ cm^−1^) calculated by the ProtParam^40^ algorithm at www.expasy.org. The concentration determination was based on an average of 3 measurements.

Data processing steps as background subtraction, scaling and merging were performed using the program PRIMUS^41^. The forward scatter, *I*(0), and the radius of gyration, *R*_g_, were obtained using the Guinier approximation^42^ or from the entire scattering profile using AUTOGNOM^43^. AUTOGNOM also provided the pair distribution function of the particle, P(*r*), and the maximum particle dimension, D_max_. The molecular mass (M_w_) was estimated from the Guinier approximation and was crosschecked by calculating the excluded volume (V_p_) of the hydrated particle from the Porod equation in AUTOPOROD^43^. Twenty *ab initio* models were reconstructed using GASBOR22i^44^ with default. The reconstructed models were aligned with SUPCOMB20^45^ and an average *ab initio* model was calculated by DAMAVER^46^. DAMFILT was used to represent the average *ab initio* model as a more compact and probable model by removing low occupancy and loosely connected dummy residues. CRYSOL^47^ was used to calculate theoretical scattering curves high-resolution X-ray structure of NCAM2 FnIII1-2 with and without His-tag. The His-tag was manually added in PyMOL. SREFLEX^22^ in default mode was used to estimate the flexibility of the NCAM2 FnIII1-2 protein.

### NCAM2 FnIII2 interaction with AMP-PCP

^13^C^15^N-NCAM2 FnIII2 was titrated with AMP-PCP, a stable ATP analogue (Sigma). From the same protein stock 2 samples were prepared: A 1 mM free form sample of FnIII2, and a 1 mM saturated form sample including 5 mM AMP-PCP. All ^15^N-HSQCs were recorded on a Varian 800 MHz spectrometer with cold probe at 298 K.

### Circular dichroism analysis of refolded FnIII domains

The far-UV CD measurements were performed using a Jasco J-815 spectropolarimeter at 20 °C in a 1 mm path length quartz cuvette from Hellma over the wavelength range 200–260 nm. A bandwidth of 1 nm and a scan speed of 20 nm/min were employed. At least 3 consecutive scans were obtained. The resulting data was smoothed and the contribution of the buffer blank was subtracted. The monitored scans were averaged.

### Surface plasmon resonance (SPR) analysis

Binding studies between NCAM2 FnIII domains and FGFR1 were conducted on a BIAcore 2000 (GE Healthcare) at 25 °C using 10 mM PBS (pH 7.4) as a running buffer. Approximately 1000 RU FGFR1β (IIIc)/Fc chimera (R&D Systems, Minneapolis, MN, USA) and 750 RU recombinant human IgG_1_ Fc (Cat. No. 110-HG, carrier-free; R&D Systems) were immobilized on a CM4 sensor chip via an amine coupling kit (BIAcore) using a flow rate of 5 μl/min. The NCAM2 domains (PBS, pH 7.4) were injected at flow rate of 20–30 μl/min. The binding curves were analyzed using the BIAevaluation version 4.1 software. A steady state affinity analysis was used to estimate *K*_*d*_.

### Synthetic peptides

Synthetic peptides derived from the sequence of the human NCAM2 FnIII1 domain included FnIII1-AB (AGVKIIELSQTTAKVS), FnIII1-CD (VDVKEVASEIWKI), and FnIII1-EF (ANNLEPNTTYEIKV). Synthetic peptides derived from the sequence of the human NCAM2 FnIII2 domain included FnIII2-AB (GQPSSGKS), FnIII2-BC (SITKQDDGGAPILEY), and FnIII2-FG (EVQITAANRLGYSEPT). All peptides were produced as dendrimers consisting of 4 monomers linked by a lysine backbone as described previously^48^ (Schafer-N A/S, Copenhagen, Denmark).

### Primary cultures of rat cerebellar granular neurons

Cerebellar granule neurons (CGNs) were prepared from 7–8 days-old Wistar rats (Charles River) as previously described^49^. CGNs were plated in 8-well uncoated Permanox® LabTek® Chamber Slides™ (Nunc®) at a density of 50.000 cells/well in Neurobasal A medium with 0.5% (v/v) Glutamax, 2% (v/v) B27 and 4 mg/ml bovine serum albumin (Invitrogen). The cells were cultured at 37 °C, 5% CO_2_ in a humidified atmosphere for 24 h before they were prepared for immunofluorescence microscopy.

No animal experiments were performed. Rats were treated in accordance with the Danish Animal Protection Act and were euthanized prior to the preparation of primary cell cultures.

### Immunofluorescence staining for *GAP-43*

CGNs were fixated in 4% (v/v) formalin, 1% methanol in PBS and immunostained with polyclonal rabbit antibodies directed against the neuron-specific protein GAP-43 (1:1000; Chemicon). The GAP-43 staining was visualized using Alexa Fluor® 488-conjugated secondary antibodies (1:1000; Invitrogen).

### Neurite outgrowth analysis

Micrographs of GAP-43-stained neurons were recorded using an inverted Zeiss Axiovert 100 microscope equipped with a 20x, 0.75 NA objective and coupled to a Zeiss, AxiocamMRm video camera (Carl Zeiss A/S, Birkeroed, Denmark). The average length of processes per neuron was estimated using a stereological approach as described^50^. For each well, 150–200 neurons were captured and analyzed.

### Sequence alignment analysis

Sequences of NCAM1- and NCAM2 FnIII1-2 from *Homo sapiens* (UniProtKB identifier P13591 and O15394), *Rattus norvegicus* (UniProtKB identifier P13596 and A0A0G2K7P9), and *Mus musculus* (UniProtKB identifier P13595 and O35136) were obtained from uniprot.org, and aligned using MUSCLE^51,52^ with default setting in Jailview (2.10.1). Sequences used can be found in SI (Table S4).

### Data availability

All data generated or analysed during this study are included in this published article (and its Supplementary Information files).

## Electronic supplementary material

Supplementary Information
