# Supplementary material for: NCAM2 Fibronectin type-III domains form a rigid structure that binds and activates the Fibroblast Growth Factor Receptor
Source: Sci Rep. 2018 Jun 12;8:8957. doi: 10.1038/s41598-018-27089-7 (PMC5997747; doi:10.1038/s41598-018-27089-7)
Supplement: Supplementary file 1 — Supplementary Information [file 41598_2018_27089_MOESM1_ESM.pdf]

NCAM2 Fibronectin type-III domains form a rigid structure that binds and activates the Fibroblast Growth Factor Receptor

Kim Krighaar Rasmussen[1,2,3,#,*], Maria Hansen Falkesgaard[2,#], Malene Winther[2], Nikolaj Kulahin Roed[2], Christine Louise Quistgaard[2], Marie Nygaard Teisen[2], Sofie Marie Edslev[2], David Leander Petersen[2], Ali Aljubouri[2], Claus Christensen[2], Peter Waaben Thulstrup[1], Leila Lo Leggio[1], Kaare Teilum[3], and Peter Schledermann Walmod[2]

1: Biological Chemistry, Department of Chemistry, University of Copenhagen, Copenhagen, Denmark

2: Laboratory of Neural Plasticity, Department of Neuroscience and Pharmacology, University of Copenhagen, Copenhagen, Denmark

3: Structural Biology and NMR Laboratory, Department of Biology, University of Copenhagen, Copenhagen, Denmark

# These authors contributed equally

*** Corresponding Author:**

Biological Chemistry, Department of Chemistry, Faculty of Science,

Universitetsparken 5, DK-2100 Copenhagen, Denmark.

E-mail: kkr@chem.ku.dk

Table S1. Structural statistics of the FnIII2 domain of NCAM2

Parameter	Value	Standard deviation
**Structural precision**[a]		
RMSD for backbone atoms (Å)	0.35	± 0.06
RMSD for heavy atoms (Å)	0.88	± 0.05
**Number of non-redundant structural restrains**		
Number of constrains	1934	
Long-range NOE [|i -j| > 4]	951	
Medium-range NOE [|i -j| ≤ 4]	146	
Sequential NOE [|i -j| =1]	491	
Intra NOE [|i –j| =0]	346	
Hydrogen bonds	21	
Dihedral angle restrain	118	
**Restrains violations**		
> 0.4 Å	None	
Dihedral angle violations >5°	None	
**RMS violation of restrains**		
NOE (Å)	0.0295	± 0.0016
Dihedral angles (°)	0.37	± 0.09
**Energies (kcal/mol) from Xplor-NIH**		
Bonds	33.32	± 2.36
Bonds angles	201.82	± 13.17
NOE	84.16	± 8.97
Dihedral angles	-686.96	± 28.61
Hydrogen bonds	3.53	± 0.65
van der Waals	154.20	± 8.13
Dihedreal bond angles	1.02	± 0.52
Improper bond angles	30.53	± 3.22
Overall	-178.38	± 35.65
**RMSDs from idealized geometry**		
Bonds lengths (Å)	0.0046	± 0.0005
Bond angles (°)	0.66	± 0.02
Improper bond angles (°)	0.48	± 0.03
**Ramachandran plot statistics**		
Most favored regions (%)	79.7	
Additionally allowed regions (%)	17.5	
Generously allowed regions (%)	2.1	
Disallowed regions (%)	0.7	

[a]RMSDs from the average for residues 3-93 in 20 structure models.

Table S2. The average total neurite length per cell for NCAM2 FnIII domains.

FnIII1	MEAN (µm)	SEM
4 µM	7.95	2.12
2 µM	6.90	1.66
1 µM	5.87	2.49
0.5 µM	5.23	1.71
0.25 µM	4.80	2.04
0.13 µM	4.10	1.33
0 µM	3.31	1.12

FnIII2	MEAN (µm)	SEM
4 µM	9.11	2.49
2 µM	6.95	2.47
1 µM	5.59	1.74
0.5 µM	5.85	2.68
0.25 µM	4.71	1.75
0.13 µM	2.95	1.28
0 µM	2.92	0.83

FnIII1-2	MEAN (µm)	SEM
4 µM	7.67	2.14
2 µM	5.67	2.03
1 µM	4.40	1.29
0.5 µM	3.08	1.13
0.25 µM	4.21	1.08
0.13 µM	3.07	0.10
0 µM	3.04	1.10

FnIII1-2 SU5402	MEAN (µm)	SEM
Coat	10.29	2.68
Coat + 100 µM SU5402	5.29	2.24
Coat + 33 µM SU5402	7.40	2.28
Coat + 11 µM SU5402	7.11	1.75
No coat	3.63	1.02
No coat + 100 µM SU5402	2.62	1.39
No coat + 33 µM SU5402	3.39	1.13
No coat + 11 µM SU5402	3.53	1.65

FnIII1-2 U0126	MEAN (µm)	SEM
Coat	8.28	1.00
Coat + 20 µM U0126	4.52	0.42
Coat + 6.6 µM U0126	7.76	1.31
Coat + 2.2 µM U0126	8.18	0.59
No coat	2.81	0.68
No coat + 20 µM U0126	3.55	0.97
No coat + 6.6 µM U0126	2.40	0.22
No coat + 2.2 µM U0126	2.24	1.28

Table S2. The average total neurite length per cell. The first three tables corresponds to concentration dependent neurite outgrowth with recombinant domains from NCAM2 (FnIII1, FnIII2 and FnIII1-2). The last to corresponds to inhibition experiments using either SU5402 or U0126 to inhibit 4 µM NCAM2 FnIII1-2 neuritogen effect. The average total lengths were **c**alculated using $L(\mu m) = (\pi * d)/2 * I$ where the equation reflects the number of intersections (I) of neurites with test lines in a counting frame with the vertical distances between the lines of d, for further details see Ronn et al. 2000[1]. Coat (4 µM FnIII1-2 recombinant protein).

Table S3. Average total neurite length per cell for peptides.

N2-FnIII1-AB	MEAN (µm)	SEM
35 µM	4.47	1.78
12 µM	12.35	1.99
4 µM	13.01	1.55
1.3 µM	13.53	1.66
0.4 µM	10.76	5.17
0.1 µM	4.08	1.45
0 µM	2.36	0.56

N2-FnIII1-CD	MEAN (µm)	SEM
38 µM	2.62	0.97
13 µM	3.33	1.11
4.2 µM	4.44	3.37
1.4 µM	2.07	1.85
0.4 µM	1.88	2.31
0.1 µM	2.98	1.33
0 µM	2.87	1.21

N2-FnIII1-EF	MEAN (µm)	SEM
35 µM	3.01	0.16
12 µM	1.87	1.61
4 µM	2.51	0.47
1.3 µM	2.35	0.98
0.4 µM	1.98	0.26
0.1 µM	3.26	1.77
0 µM	3.57	1.06

N2-FnIII2-AB	MEAN (µm)	SEM
72 µM	4.85	3.38
24 µM	5.89	4.23
8 µM	4.31	2.33
2.7 µM	5.55	6.29
0.9 µM	4.19	1.90
0.3 µM	6.26	5.48
0 µM	6.65	3.77

N2-FnIII2-BC	MEAN (µm)	SEM
36 µM	7.43	4.77
12 µM	10.84	2.75
4 µM	9.88	2.57
1.3 µM	11.48	5.64
0.4 µM	8.25	5.74
0.15 µM	7.20	3.46
0 µM	5.89	2.83

N2-FnIII2-FG	MEAN (µm)	SEM
33 µM	5.49	2.68
11 µM	1.97	0.24
3.7 µM	1.45	0.15
1.2 µM	2.05	1.18
0.4 µM	2.88	1.12
0.14 µM	2.70	2.24
0 µM	2.70	2.00

N2-FnIII1-AB SU5402	MEAN (µm)	SEM
Coat	18.07	14.17
Coat + 100 µM SU5402	12.19	9.38
Coat + 33 µM SU5402	12.79	10.62
Coat + 11 µM SU5402	11.94	11.60
No coat	8.27	6.63
No coat + 100 µM SU5402	8.43	6.29
No coat + 33 µM SU5402	8.76	5.39
No coat + 11 µM SU5402	6.22	5.74

N2-FnIII1-AB U0126	MEAN (µm)	SEM
Coat	6.91	0.58
Coat + 20 µM U0126	5.04	1.55
Coat + 6.6 µM U0126	4.64	2.43
Coat + 2.2 µM U0126	3.05	2.94
No coat	2.65	0.04
No coat + 20 µM U0126	4.45	1.21
No coat + 6.6 µM U0126	3.18	1.70
No coat + 2.2 µM U0126	1.85	0.42

Table S3. The average total neurite length per cell. Average total neurite lengths were calculated as in Table S2. The first six tables correspond to concentration dependent neurite outgrowth with peptides (FnIII1 AB, - CD, - EF and FnIII2 AB, - BC, - FG). The last two corresponds to inhibition experiments performed with FnIII1-AB.

Table S4. Sequences used for alignment

NCAM2	Human (O15394)	496-PSSPY GVKIIELSQT TAKVSFNKPD SHGGVPIHHY QVDVKEVASE IWKIVRSHGV QTMVVLNNLE PNTTYEIRVA AVNGKGQGDY SKIEIFQTLP VREPSPPSIH GQPSSGKSFK LSITKQDDGG APILEYIVKY RSKDKEDQWL EKKVQGNKDH IILEHLQWTM GYEVQITAAN RLGYSEPTVY EFSMPPKPNI IKD-693
	Rattus Norvegicus (A0A0G2K7P9)	495-PSSPR GVKIIELSQT TAKISFNKPE SHGGVPIHHY QVDVKEVTSE TWKIVRSHGV QTTVVLSSLE PNTTYEVRVA AVNGKGQGDY SKIEIFQTLP VREPSPPSIH GQPSSGKSFK ISITKQDDGG APILEYIVKY RSKDKEDQWL EKKVQGNKDH IILEHLQWTM GYEVQITAAN RLGYSEPTVY EFSMPPKPNI IKD-692
	Mus musculus (O35136)	496-PSSPH GVKIIELSQT TAKISFNKPE SHGGVPIHHY QVDVKEVASE TWKIVRSHGV QTMVVLSSLE PNTTYEIRVA AVNGKGQGDY SKIEIFQTLP VREPSPPSIH GQPSSGKSFK ISITKQDDGG APILEYIVKY RSKDKEDQWL EKKVQGNKDH IILEHLQWTM GYEVQITAAN RLGYSEPTVY EFSMPPKPNI IKD-693
NCAM1	Human (P13591)	510-PSSPS IDQVEPYSST AQVQFDEPEA TGGVPILKYK AEWRAVGEEV WHSKWYDAKE ASMEGIVTIV GLKPETTYAV RLAALNGKGL GEISAASEFK TQPVQGEPSA PKLEGQMGED GNSIKVNLIK QDDGGSPIRH YLVRYRALSS EWKPEIRLPS GSDHVMLKSL DWNAEYEVYV VAENQQGKSK AAHFVFRTSA QP-706
	Rattus Norvegicus (P13596)	511-PSSPS IDRVEPYSST AQVQFDEPEA TGGVPILKYK AEWKSLGEEA WHSKWYDAKE ANMEGIVTIM GLKPETRYAV RLAALNGKGL GEISAATEFK TQPVREPSAP KLEGQMGEDG NSIKVNLIKQ DDGGSPIRHY LVKYRALASE WKPEIRLPSG SDHVMLKSLD WNAEYEVYVV AENQQGKSKA AHFVFRTSAQ P-706
	Mus musculus (P13595)	501-PSSPS IDRVEPYSST AQVQFDEPEA TGGVPILKYK AEWKSLGEES WHFKWYDAKE ANMEGIVTIM GLKPETRYSV RLAALNGKGL GEISAATEFK TQPVRPSAPK LEGQMGEDGN SIKVNLIKQD DGGSPIRHYL VKYRALASEW KPEIRLPSGS DHVMLKSLDW NAEYEVYVVA ENQQGKSKAA HFVFRTSAQP -696

Table S4. UniProtKB identifier are given in parentheses

Figure S1

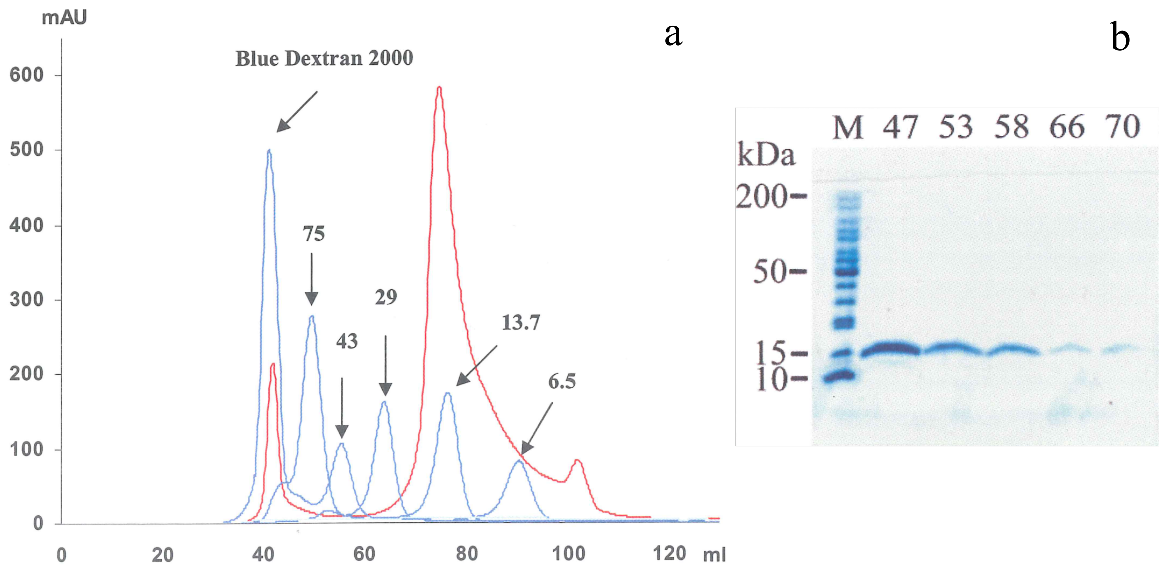

Figure S1. Purity of $^{13}C^{15}N$ labelled NCAM2 FnIII2 domain expressed in yeast. The quality of the NCAM2 FnIII2 domain, expressed in yeast and used for structural studies using NMR, was verified from SEC (Fig S1. a) and SDS-PAGE (Fig S1. b). $^{13}C^{15}N$-FnIII2 (red, curve) purified on a Superdex75 PrepGrad (GE Healthcare) calibrated with GE healthcare low molecular weight standards (blue curve) (Blue Dextran 2000, Conalbumin 75 -, Ovalalumin 43 -, Carbonic Anhydrase 29 -, Ribonuclease A 13.7 – and Aprotinin 6.5 kDa. The first peak in the $^{13}C^{15}N$-FnIII2 chromatogram eluted at ~41 mL presumably corresponds to aggregated protein, and thus was discarded. The second asymmetric and most prominent peak corresponds to monomeric $^{13}C^{15}N$-FnIII2. To confirm purity the asymmetric peak of $^{13}C^{15}N$-FnIII2 was analyzed by 8-25 % gradient SDS-PAGE using the PhastSystem™ using sample applicator 6/4 (6 samples / 4 uL) (b). Fraction 47, 53, 58, 66 and 70 corresponds to 79, 85, 91, 98 and 102 ml elution volume, respectively.

Figure S2

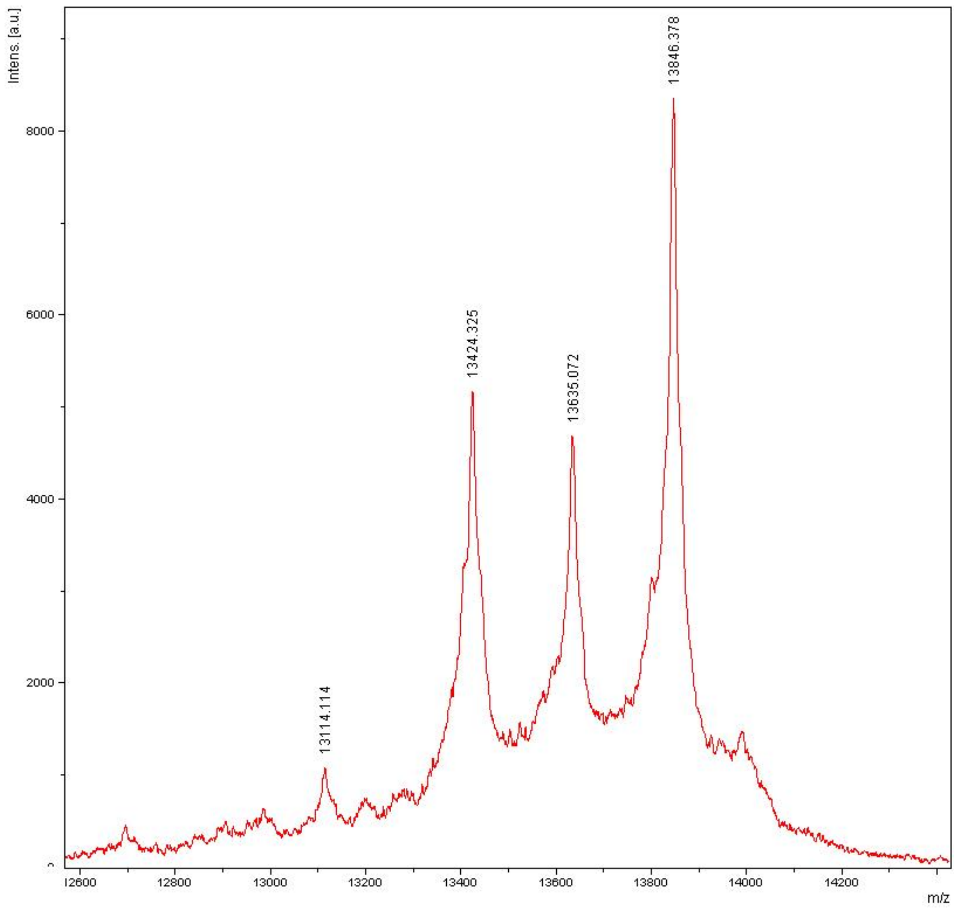

Figure S2. MALDI-TOF spectrum of NCAM2 FnIII2 produced in P. pastoris. The spectrum shows that FnIII2 exists in 3 variants. The variation originates from insufficient cleavage of N-terminal signal peptide (EAEA), due to inefficient Ste13 enzyme activity. The peaks correspond to completely, partially, and uncleaved signal peptide.

Figure S3

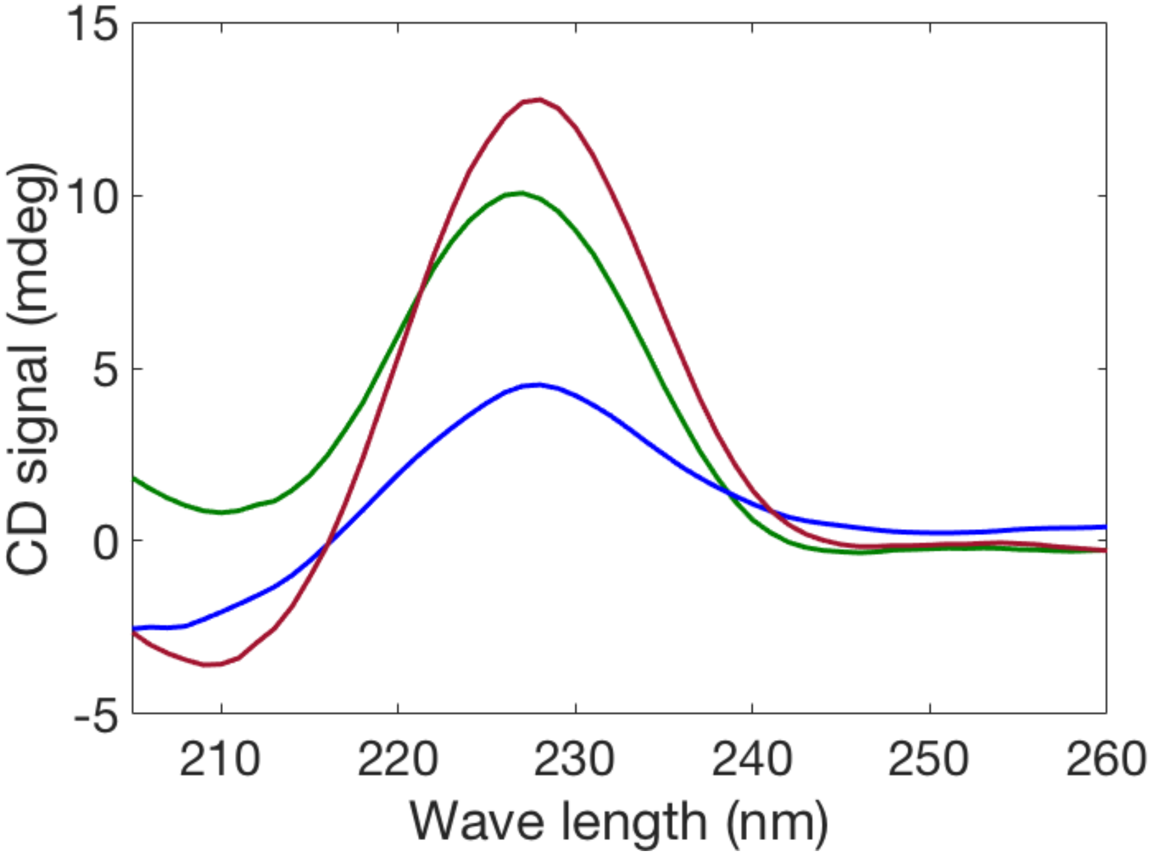

Figure S3. CD spectra of NCAM2 FnIII domains. The FnIII domains were expressed in *E. coli* (BL21) and refolded from inclusion bodies. CD spectra of all NCAM2 FnIII proteins (FnIII1 [green], FnIII2 [blue], and FnIII1-2 [red]) had the characteristic positive signal for folded FnIII domain at ~227 nm[2].

.

Figure S4

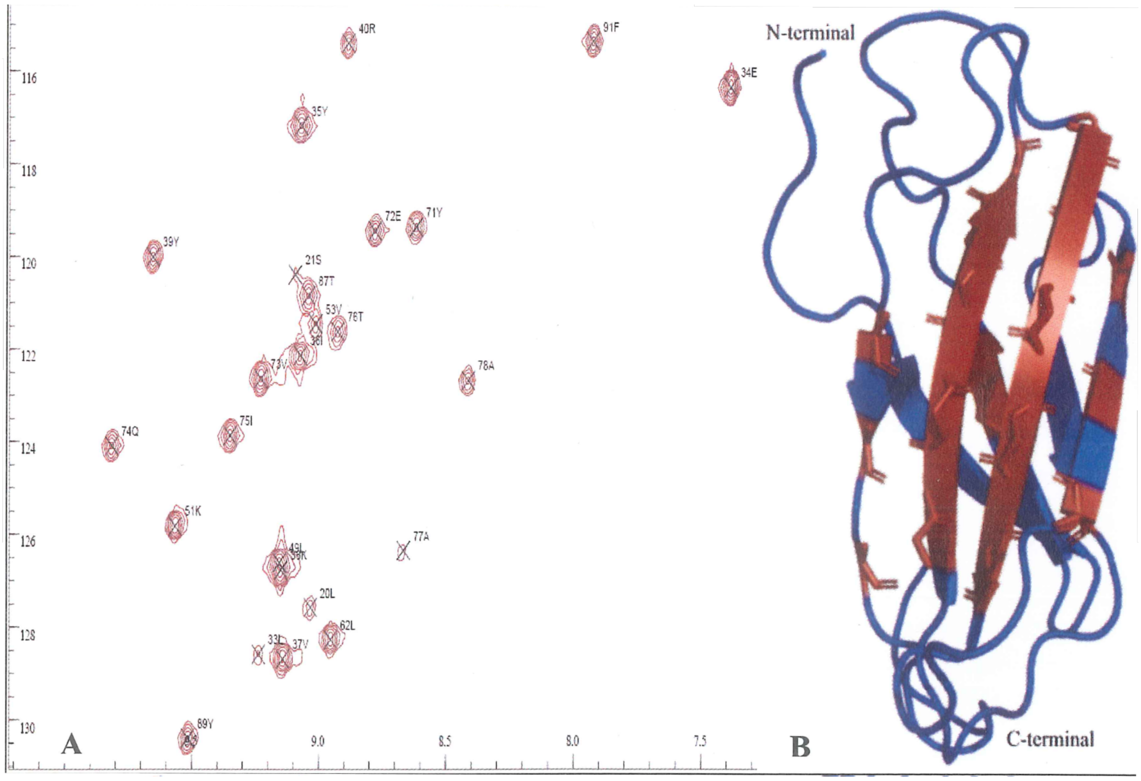

Figure S4. **Slow exchanges residues involved in hydrogen binding.** Representation of a ^{15}N-HSQC recorded 60 min. after ^{1}H/^{2}H exchanges (a). Residues involved in hydrogen bonds are colored brown and mapped on the NCAM2 FnIII2 structure (b).

Figure S5

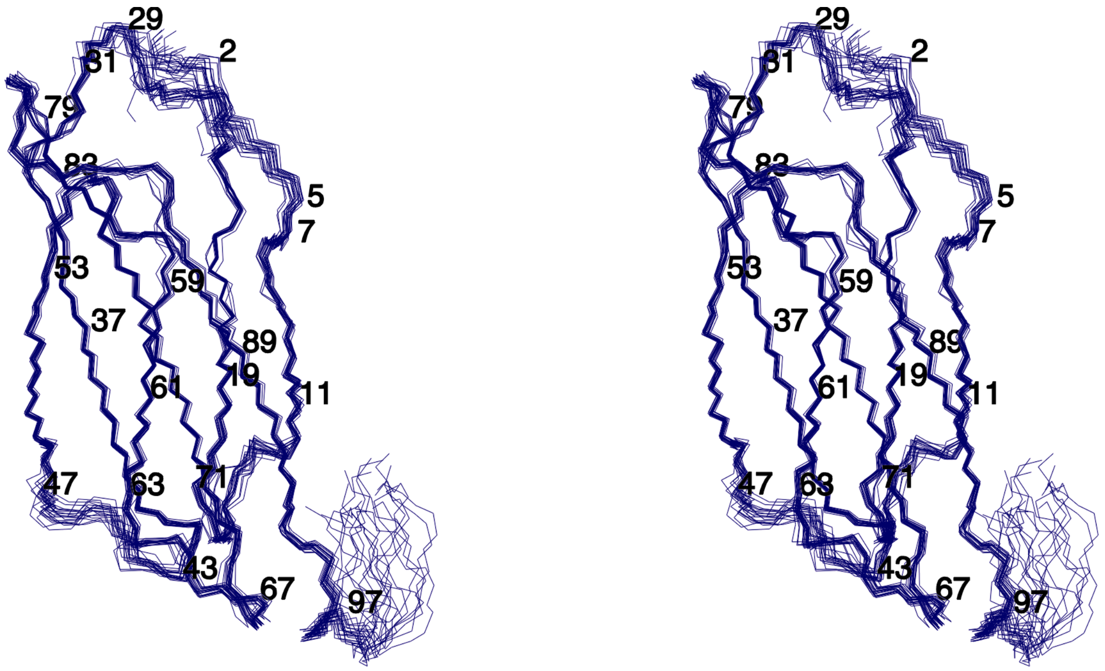

Figure S5. Structure models of the NCAM2 FnIII2 domain. Stereo view of an overlay of the backbone of 20 superimposed structural models of the FnIII2 domain. Numbers indicate randomly selected amino acids to facilitate orientation through the backbone. From 100 structural models the 20 structural models with the lowest energies were selected to represent the structural model of NCAM2 FnIII2 (PDB code: 2kbg). The obtained model contains no individual structures with NOE constrain violations above 0.4 Å or bond angle violations above 5°. A summary of the structural statistics is given in Table S1.

Figure S6

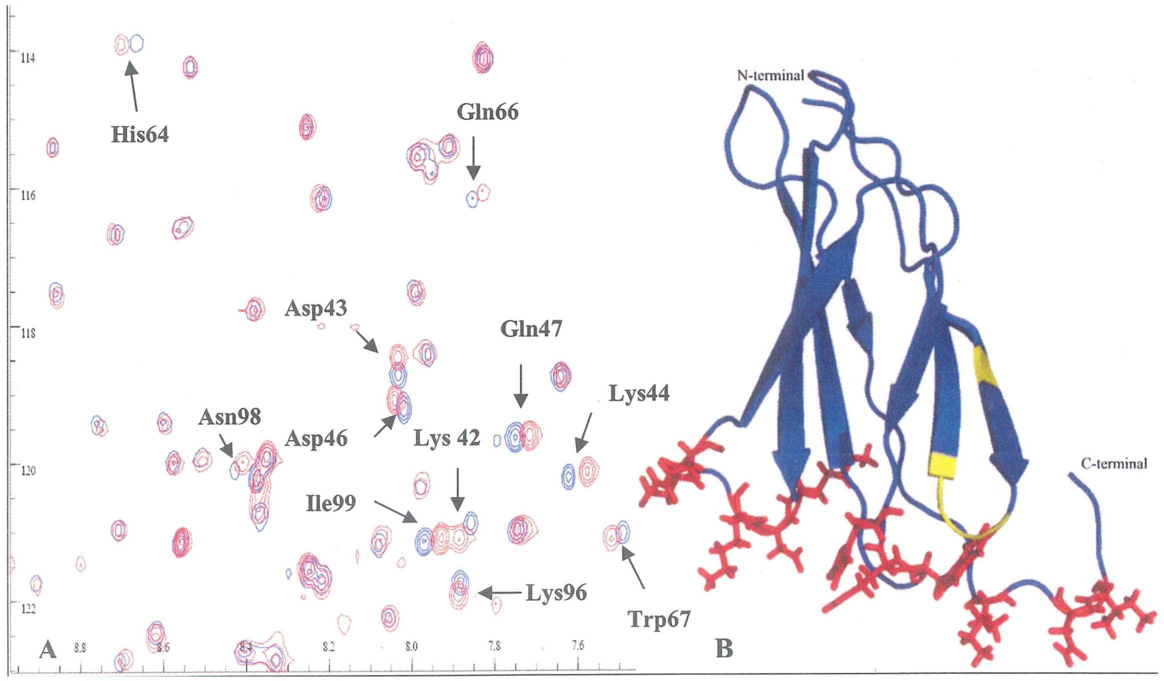

Figure S6. AMP-PCP titration data. The figure shows end point HSQC experiments of NCAM2 FnIII2 with (red) or without (blue) AMP-PCP. AMP-PCP was used to investigate if NCAM2 FnIII2 could bind AMP-PCP via the Walker A motif (yellow on structure). Only small chemical shift changes were observed (colored red on structure), and none of those were located in the Walker A motif.

Figure S7

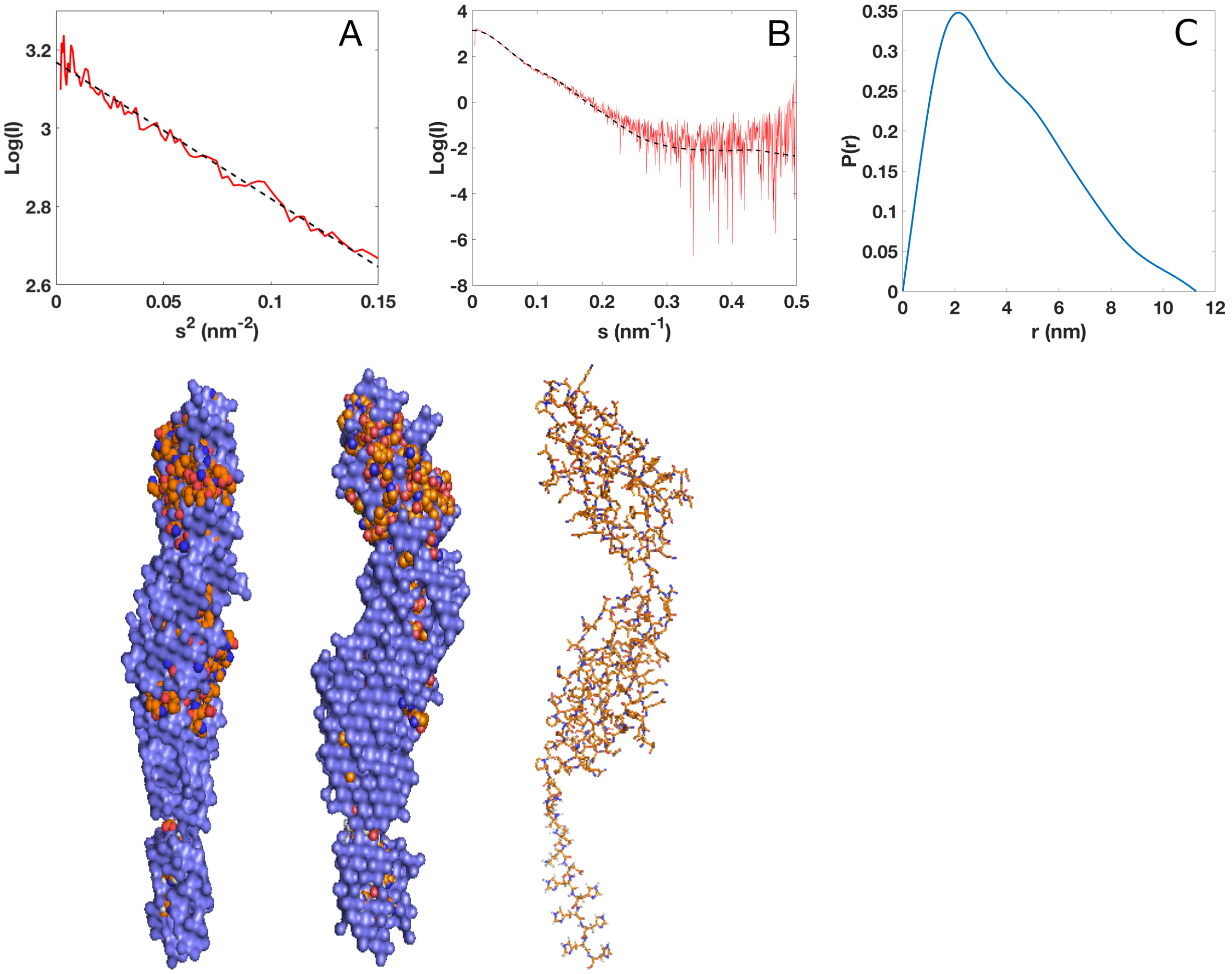

Figure S7. Top: SAXS data for NCAM2 FnIII1-2. Experimental SAXS data (A, B red) with linear fit to Guinier region (A, black line) and fit to the theoretical scattering curve (B, black line) calculated from the structure of the NCAM2 FnIII1-2 His-tag. Bottom: Reconstructed *ab initio* models (blue surface), calculated from distance distribution curve (C), and overlaid with crystal structure of NCAM2 FnIII1-2 including a His-tag (surface and lines) manually added.

Figure S8

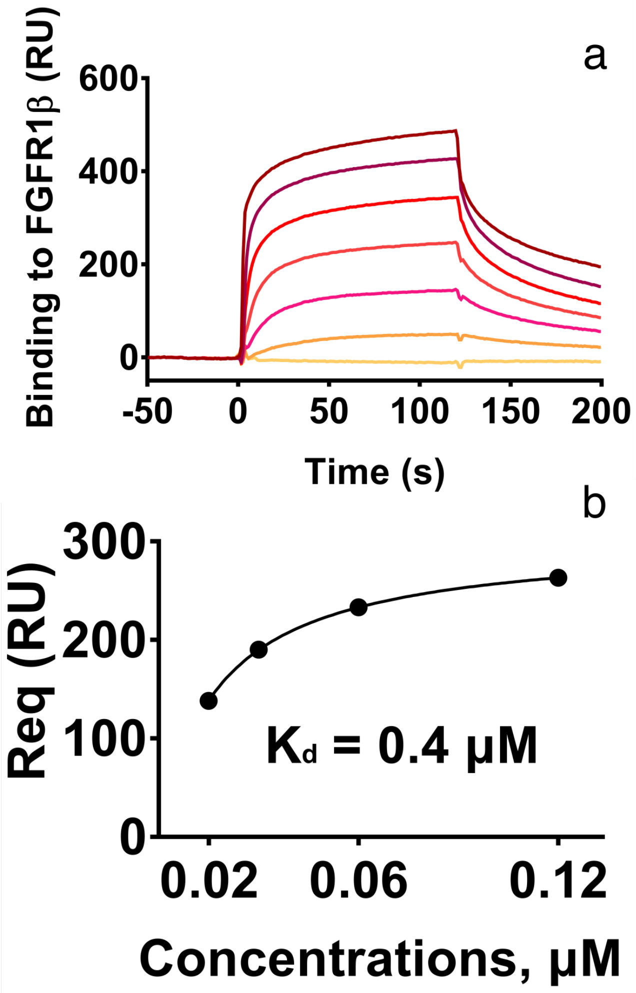

Figure S8. Estimation of K_d for NCAM2-FnIII1-2 and FGFRβ. The K_d between NCAM2-FnIII1-2 expressed in *P.pastoris* and FGFR was estimated using surface plasmon resonance. Except for the flow time (200 second) the protocol was identical to the description in material and methods. Increasing concentration of analyte (NCAM2) were flowed over the immobilized binding partner FGFRβ (a). Steady state fitting gave a K_d = 0.4 μM with a χ^2 value of of the fit = 0.061 (b). Comparing the K_d's obtained from recombinant bacterial expressed and yeast expressed protein shows that the Kd are reproducibly in the μM range.

Figure S9

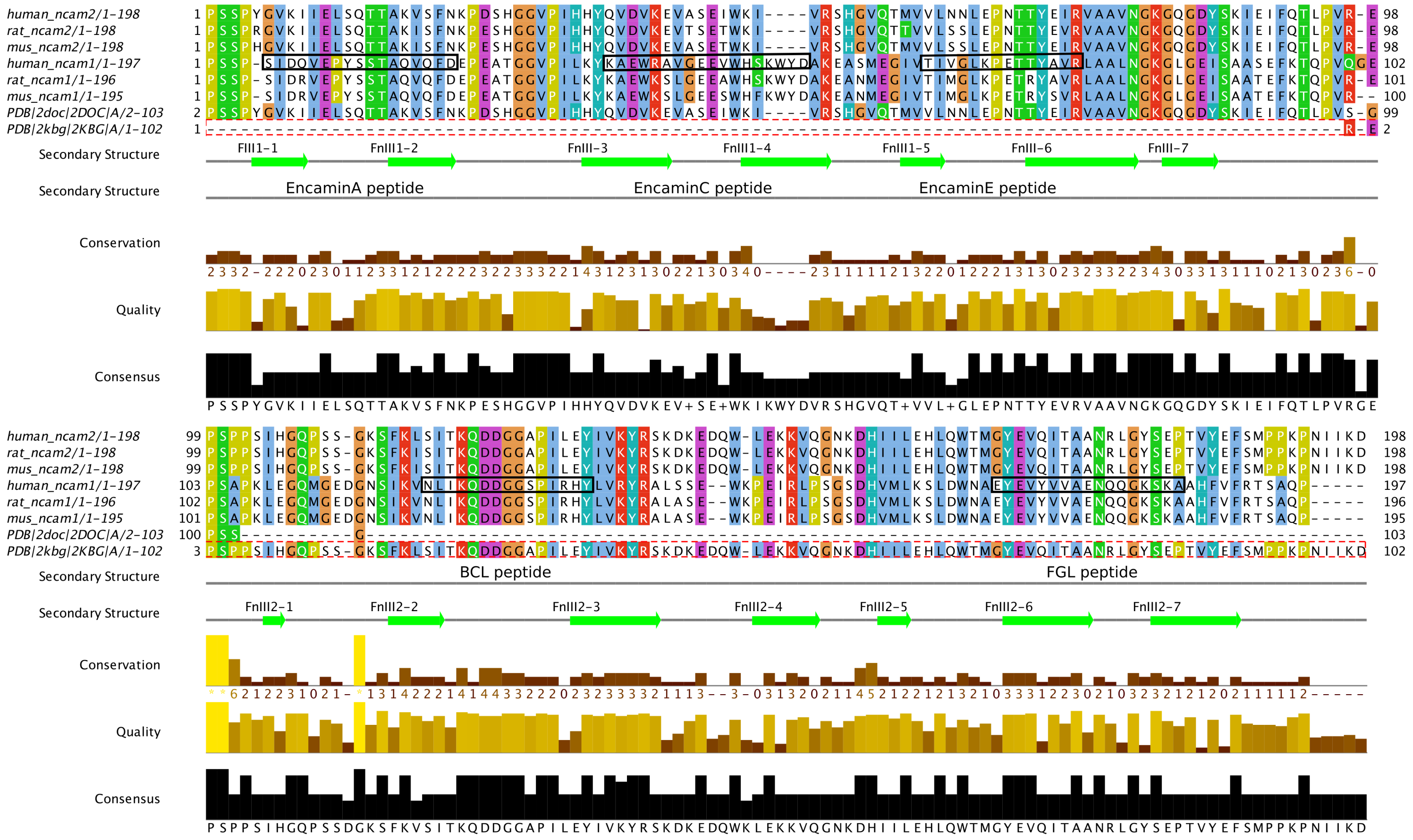

Figure S9. Alignment of the primary structures of the NCAM1 and NCAM2 FnIII domains. Protein sequences from selected species (human, rat, and mouse) were aligned to investigate conservation across species. Sequences of biologically active peptides derived from NCAM1 (EncaminA, -C, -E, BCL, and FGL) are framed.

References:

1. Rønn, L. C. *et al.* A simple procedure for quantification of neurite outgrowth based on stereological principles. *J. Neurosci. Methods* **100,** 25–32 (2000).
2. Yang, L. *et al.* Expression, refolding and spectroscopic characterization of fibronectin type III (FnIII)-homology domains derived from human fibronectin leucine rich transmembrane protein (FLRT)-1, -2, and -3. *PeerJ* **5,** e3550 (2017).
